# Quantum mechanics-based multitensor AI/ML uniquely able to discover, validate, and interpret predictors from small-cohort noisy high-dimensional multiomic data

**DOI:** 10.1063/5.0305656

**Published:** 2026-06-22

**Authors:** Orly Alter, Elizabeth Newman, Sri Priya Ponnapalli, Jessica W. Tsai

**Affiliations:** 1 Scientific Computing and Imaging Institute, University of Utah, Salt Lake City, Utah 84112, USA; 2 Department of Bioengineering, Department of Human Genetics, and Huntsman Cancer Institute, University of Utah, Salt Lake City, Utah 84112, USA; 3 Prism AI Therapeutics, Inc., Salt Lake City, Utah 84103, USA; 4 Department of Mathematics, Tufts University, Medford, Massachusetts 02155, USA; 5 Scale AI, Inc., San Francisco, California 94103, USA; 6 Cancer and Blood Disease Institute, Children’s Hospital of Los Angeles, Los Angeles, California 90027, and Department of Pediatrics, Keck School of Medicine of the University of Southern California, Los Angeles, California, 90033, USA

## Abstract

Prediction in medicine remains limited. Previously, by using our “comparative spectral decompositions” of two matrices and, separately, two third-order tensors, we demonstrated accurate, precise, actionable, and interpretable tumor whole-genome and, separately, whole-transcriptome predictors—of patients’ survival, treatment responses, and drug targets—in different cancers. Here, we introduce a unified framework that generalizes these exact and structure-preserving algorithms to multiple tensors of any order to model real-world data that measure multiple aspects of interrelated phenomena. We prove properties (e.g., existence and uniqueness) and define metrics (e.g., the “multitensor joint Shannon entropy” and the “multitensor comparative angular distance”) necessary to derive, test, and explain a model. We highlight the novel connection to the quantum mechanical concept of “entanglement” in addition to that of “superposition.” We illustrate the framework in the discovery and validation of two novel predictors in neuroblastoma—each with three entangled representations—in the tumor and blood genomes and tumor transcriptome, where the result of the measurement of any one representation approximately determines the results of the measurements of the other two. Finally, we show that in every representation, the two predictors combined are consistently more accurate than the best standard-of-care biomarker (i.e., the one-gene test for tumor *MYCN* amplification), and we interpret them in terms of known and new disease mechanisms and drug targets.

## INTRODUCTION

Two decades after the completion of the human genome project,^1^ and one decade after the first U.S. Food and Drug Administration (FDA) authorization for next-generation sequencing,^2^ prediction in medicine remains limited.^3^ This is because most artificial intelligence and machine learning (AI/ML) approaches still find real-world multiomic data extremely difficult to correctly model and interpret.

Multiomic data (e.g., from the same sets of patients and sequencing platforms) are of vastly diverse types (e.g., tumor or blood DNA or tumor RNA sequence counts). This results in a complex structure that generally takes a multitensor form of multiple layers, of two or more dimensions each, where one-to-one mapping exists among all of the sample dimensions (e.g., the patients and platforms) but not among the feature dimensions (e.g., the specific tumor or blood DNA or tumor RNA sequences).

Moreover, the data are noisy. Systematic batch effects^4^ and normal demographic variations—few of which can be anticipated—mask disease-specific patterns, while unavoidable—sometimes by design—imbalances and hard-to-quantify or unavailable labels obscure distinctions among clinical subpopulations.

Finally, the data are skinny^5–8^ (i.e., with many orders of magnitude fewer samples than features). For example, clinical trials regularly enroll only 20–100 patients.^9–11^

However, most AI/ML methods do not guarantee a solution and fail to converge to a model when the data are skinny and noisy, especially when the modeling is unsupervised. For example, a recent self-supervised large language model (LLM) of the genome of the COVID-19 virus required four orders of magnitude more samples than features.^12^ For the 3B-nucleotide human genome, this would roughly be 33T patients.

Moreover, most AI/ML models, when they exist, are not unique. This makes their interpretation, in terms of the unique phenomena that underlie the data and their unique interrelations, tenuous.

Finally, most AI/ML computations are lossy. Previously, we introduced the exact and structure-preserving (i.e., lossless) “comparative spectral decompositions” for two matrices, multiple matrices, and two third-order tensors. We proved that these blind source separation algorithms^13–15^—from the reformulated generalized singular value decomposition (GSVD)^16^ (also Refs. 17–21) to the higher-order GSVD (HO GSVD)^22,23^ (also Refs. 24–28) and the tensor GSVD^29,30^ (also Refs. 31–35)—extend the properties of the singular value decomposition (SVD) of one matrix^36,37^ (also Refs. 38–48) and the SVD-computed pseudoinverse projection^49^ (also Refs. 50–52). We defined metrics to derive, test, and explain a GSVD, HO GSVD, or tensor GSVD model and demonstrated correct prediction from multiomic data.

For example, the GSVD was demonstrated in the unsupervised modeling of tumor and blood whole genomes of 85 astrocytoma patients from The Cancer Genome Atlas (TCGA).^53^ Interpretation showed that the modeling blindly removed batch effects, separated normal demographic variations, and discovered a disease-specific genome-wide pattern of DNA copy-number alterations (CNAs), which was then used to derive a predictor. Both the predictor and the modeling were computationally validated in federated studies of mutually exclusive sets of 59–251 patients. The modeling repeatedly discovered a representation of the predictor in every study across astrocytoma grades II, III, and IV (i.e., glioblastoma, or GBM) patients. The predictor was additionally experimentally validated in a clinical trial of 79 GBM patients, initially retrospectively, and, in a four-year follow up, also prospectively.^54,55^ At 75%–95% concordance with survival in all cohorts, the predictor is more accurate than all standard-of-care indicators. At 100% reproducibility among Complete Genomics, Illumina, and Ultima Genomics whole-genome sequencing (WGS) and >99% when including Affymetrix and Agilent DNA microarrays, the predictor is also the most precise. Recent functional genomic experiments validated both a predicted drug target and the predicted tumors’ differential responses to its targeting^10,56^ (also Ref. 57). This solved the 75-year-old problem of correctly predicting GBM patients’ survival, treatment responses, and drug targets from their tumors’ whole genomes, where, since 1950, the best standard-of-care indicator has remained age.^58–61^

**FIG. 1.**
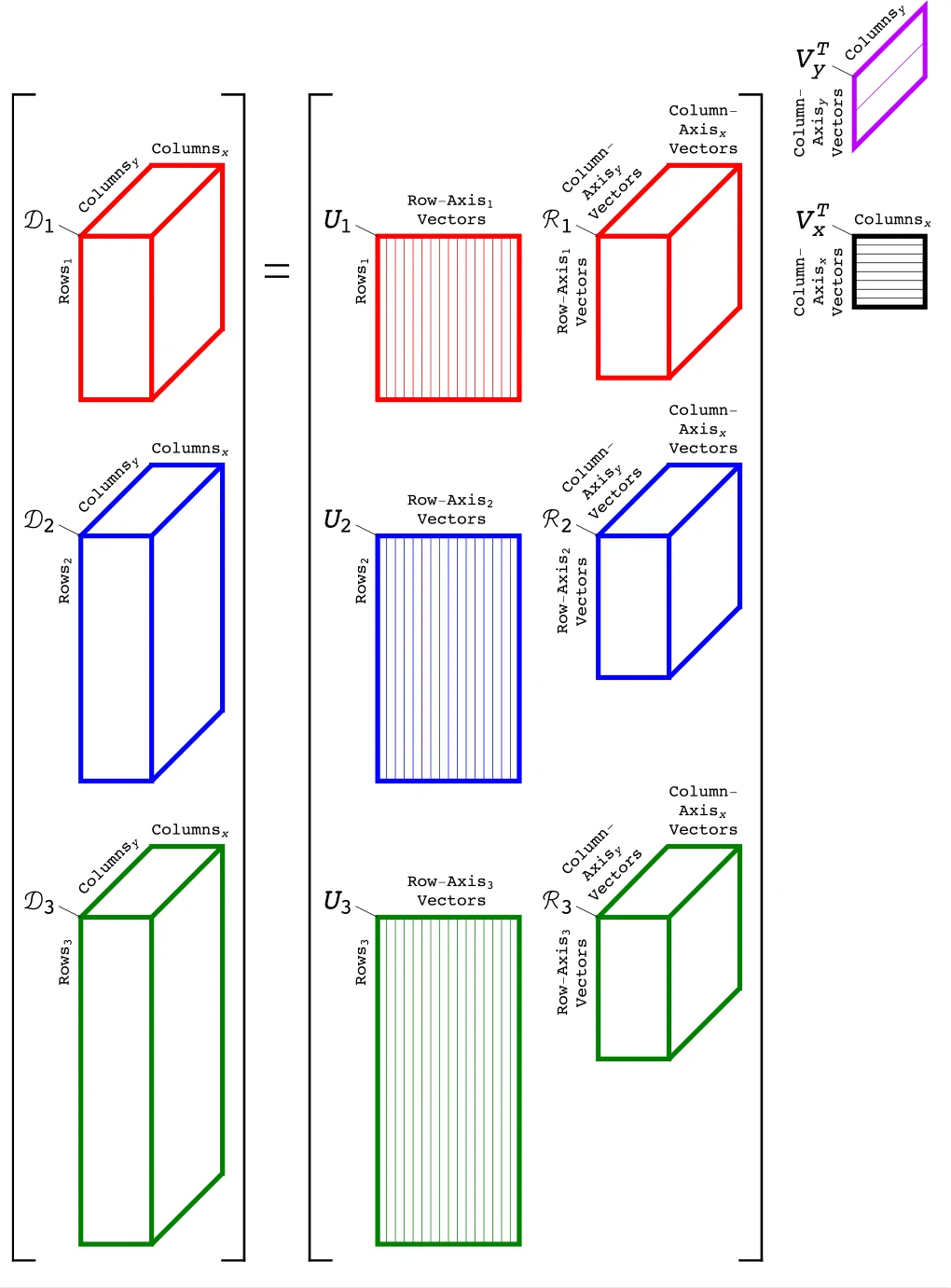
The unified framework: The multitensor comparative spectral decomposition of Eqs. (1), (2), and (9)–(13).

**FIG. 2.**
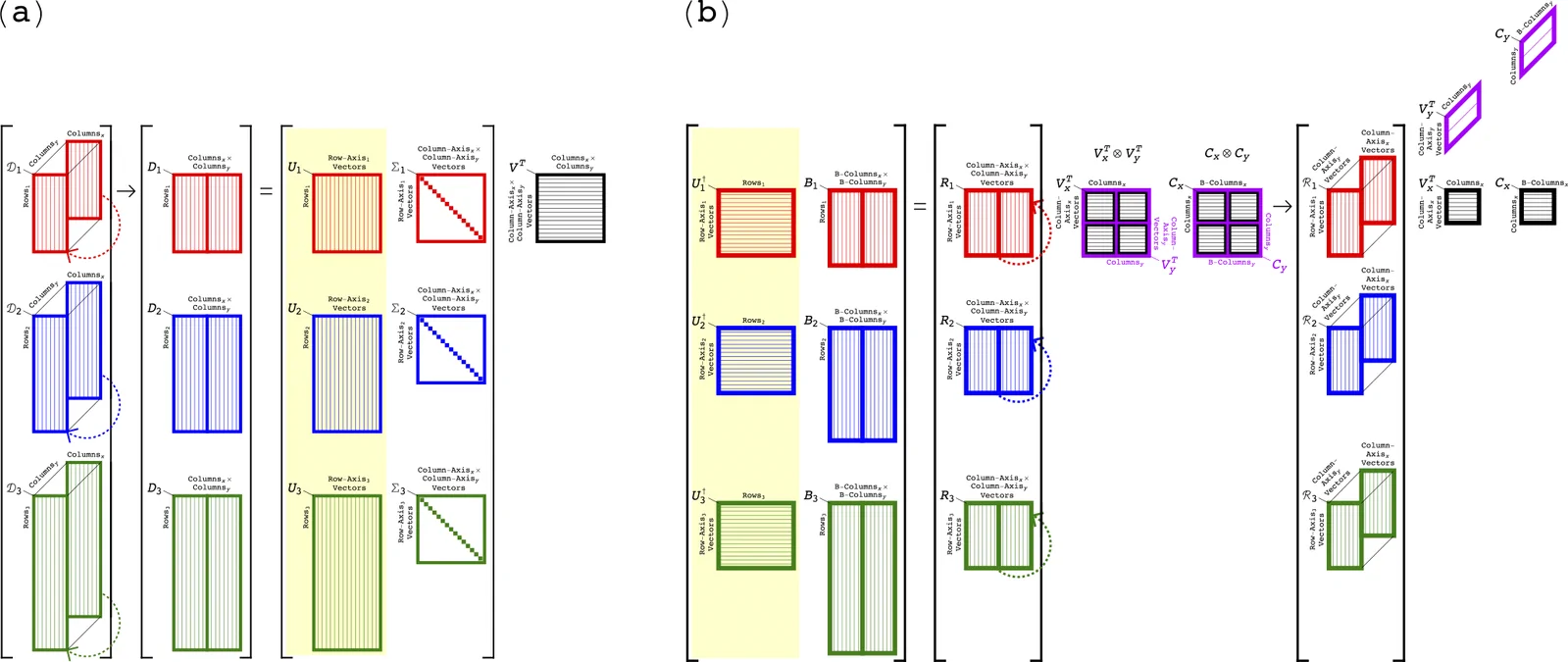
Model discovery and validation within the unified framework. (a) Unsupervised discovery via the tensor HO GSVD extends the SVD, as in Eqs. (3) and (5)–(8). (b) Semisupervised validation via the SVD-computed pseudoinverse projection of Eq. (4).

Here, we introduce a unified framework that generalizes the lossless comparative spectral decompositions to the multitensor structure (Figs. 1 and 2). We prove that the framework always converges to a model that is almost always unique and define the necessary metrics.

**FIG. 3.**
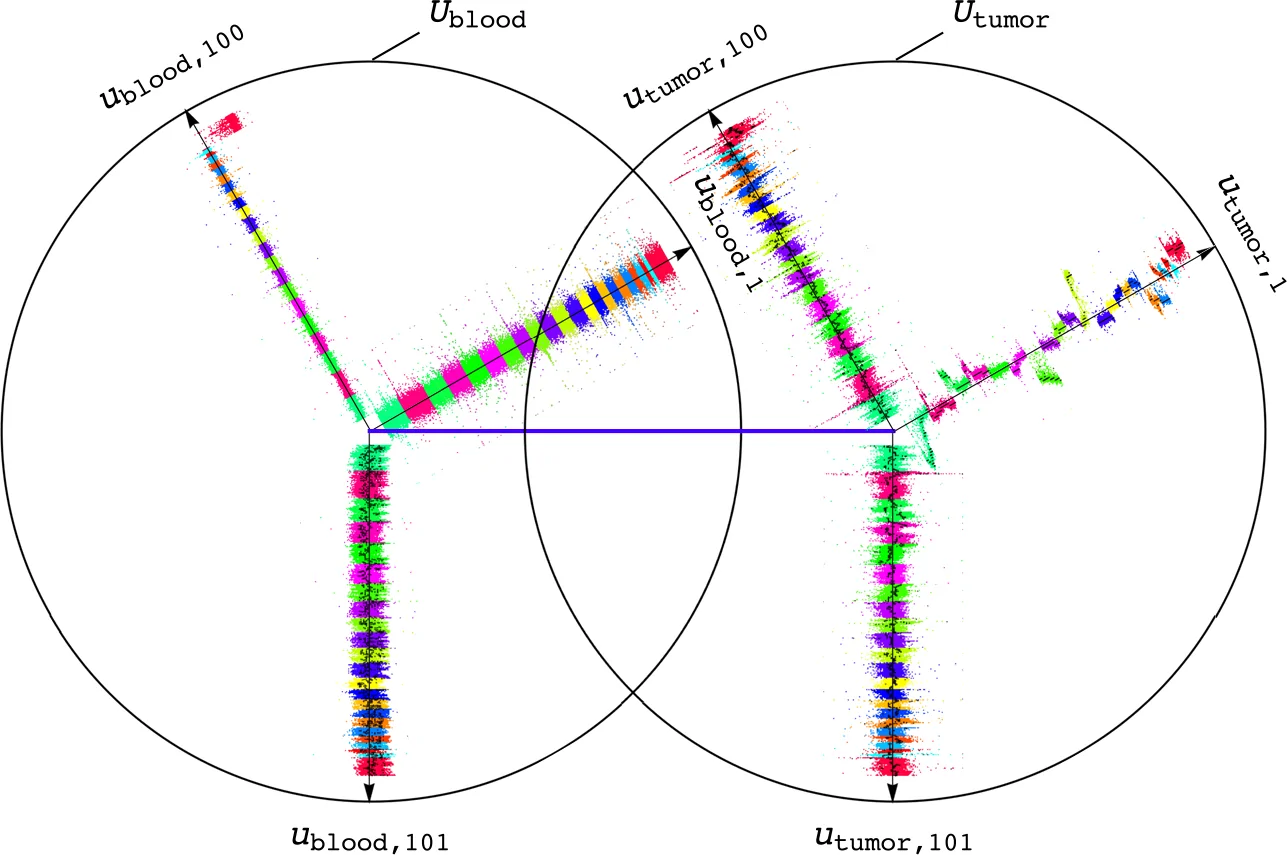
Novel connection to the quantum mechanical concept of entanglement. The framework rewrites the tumor and blood genomes of a patient $\left|d_{\mathrm{tumor}}\right\rangle ⊗\left|d_{\mathrm{blood}}\right\rangle$ as a superposition of three phenotypes represented by two entangled patterns each: $\left|u_{\mathrm{tumor},\;1}\right\rangle ⊗\left|u_{\mathrm{blood},\;1}\right\rangle +\left|u_{\mathrm{tumor},\;100}\right\rangle ⊗\left|u_{\mathrm{blood},\;100}\right\rangle +\left|u_{\mathrm{tumor},\;101}\right\rangle ⊗\left|u_{\mathrm{blood},\;101}\right\rangle$, such that the result of the measurement of either genome approximately determines the result of the measurement of the other one, as in Eqs. (14) and (15).

We highlight the framework’s novel connection to the quantum mechanical concept of entanglement in addition to that of superposition. The modeling rewrites each set of one-to-one mapped samples (e.g., from the same patient) as a superposition of states (e.g., phenotypes), each represented by a set of entangled patterns, one per data layer (e.g., a tumor or blood genotype or a tumor “transcriptype”) (Fig. 3). The patterns are used to derive predictors (e.g., of a patient’s phenotype). That the multiple patterns representing a state are entangled means that the result of the measurement of any one representation approximately determines the results of the measurements of the other ones (e.g., knowing a patient’s tumor genotype means—in the context of their phenotype—also approximately knowing their blood genotype and tumor transcriptype), and the prediction (e.g., of the patient’s phenotype) based upon any one data layer is consistent with that based upon all the other ones.

**FIG. 4.**
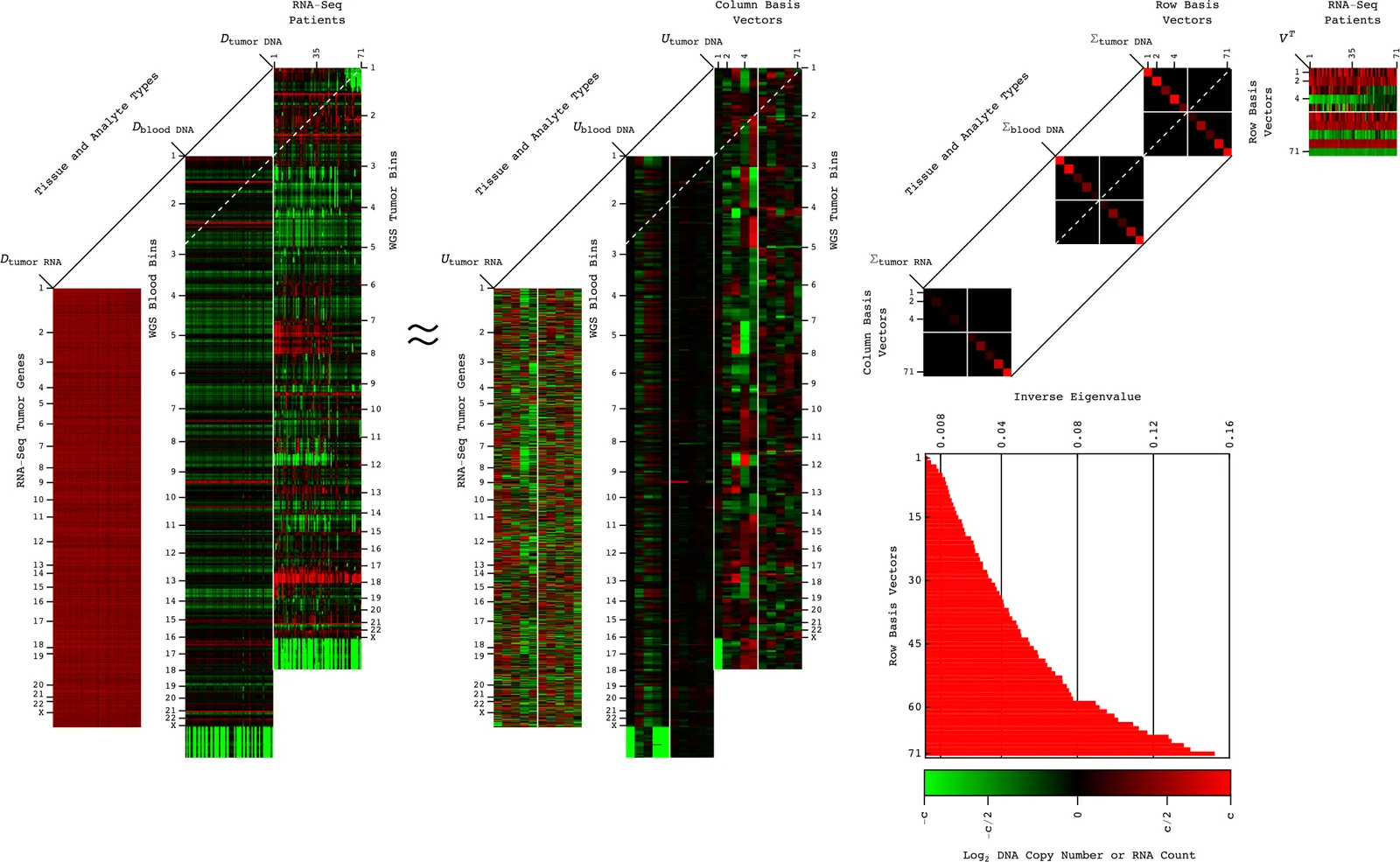
Unsupervised model discovery from real-world, skinny, and noisy data at the multimatrix limit. The HO GSVD of Eqs. (5)–(8) and (13) of the X=71 NBL patient-matched tumor and blood WGS and tumor RNA-Seq profiles, comprising $Z_{1}=2,\;831\;960$, $Z_{2}=2,\;831\;959$, and $Z_{3}=15\;393$ tumor and blood genomic and tumor transcriptomic features, respectively, explicitly showing the first through the 5th and the 67th through 71st: rows of the shared factor matrix $V^{T}$, columns of the data layer-specific factor matrices $U_{n}$, and diagonal elements in the core matrices $\Sigma_{n},n=1,\;2,\;3$. The contrasts of overabundance (red) and underabundance (green) of the log_2_ DNA copy numbers relative to the autosomal median (black) in each profile for layers $D_{1}$ and $D_{2}$ and matrices $U_{1}$ and $U_{2}$ are c=1.2 and 750, respectively. The corresponding contrasts of log_2_ RNA counts in $D_{3}$ and $U_{3}$ are 5 × 10^−2^ and 100. The contrasts in $\Sigma_{n}$ and $V^{T}$ are 5 × 10^−4^ and 5, respectively. The inset bar chart depicts the inverse eigenvalues.

**FIG. 5.**
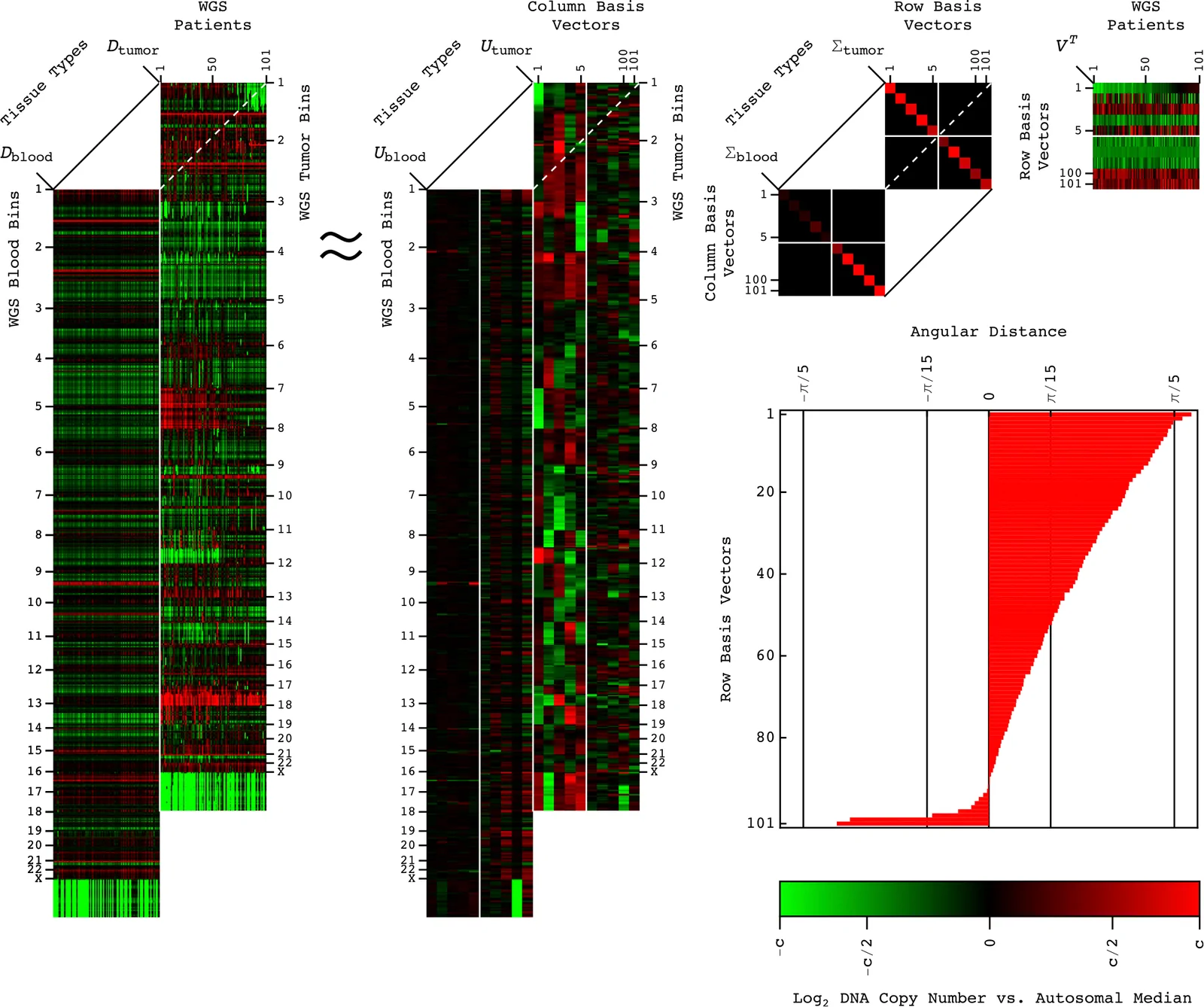
Unsupervised discovery from real-world data at the two-matrix limit. The GSVD of the WGS profiles of 101 NBL patient-matched tumor and blood samples, comprising 2 831 960 and 2 831 959 genomic features, respectively. The inset bar chart depicts the GSVD angular distances.

**FIG. 6.**
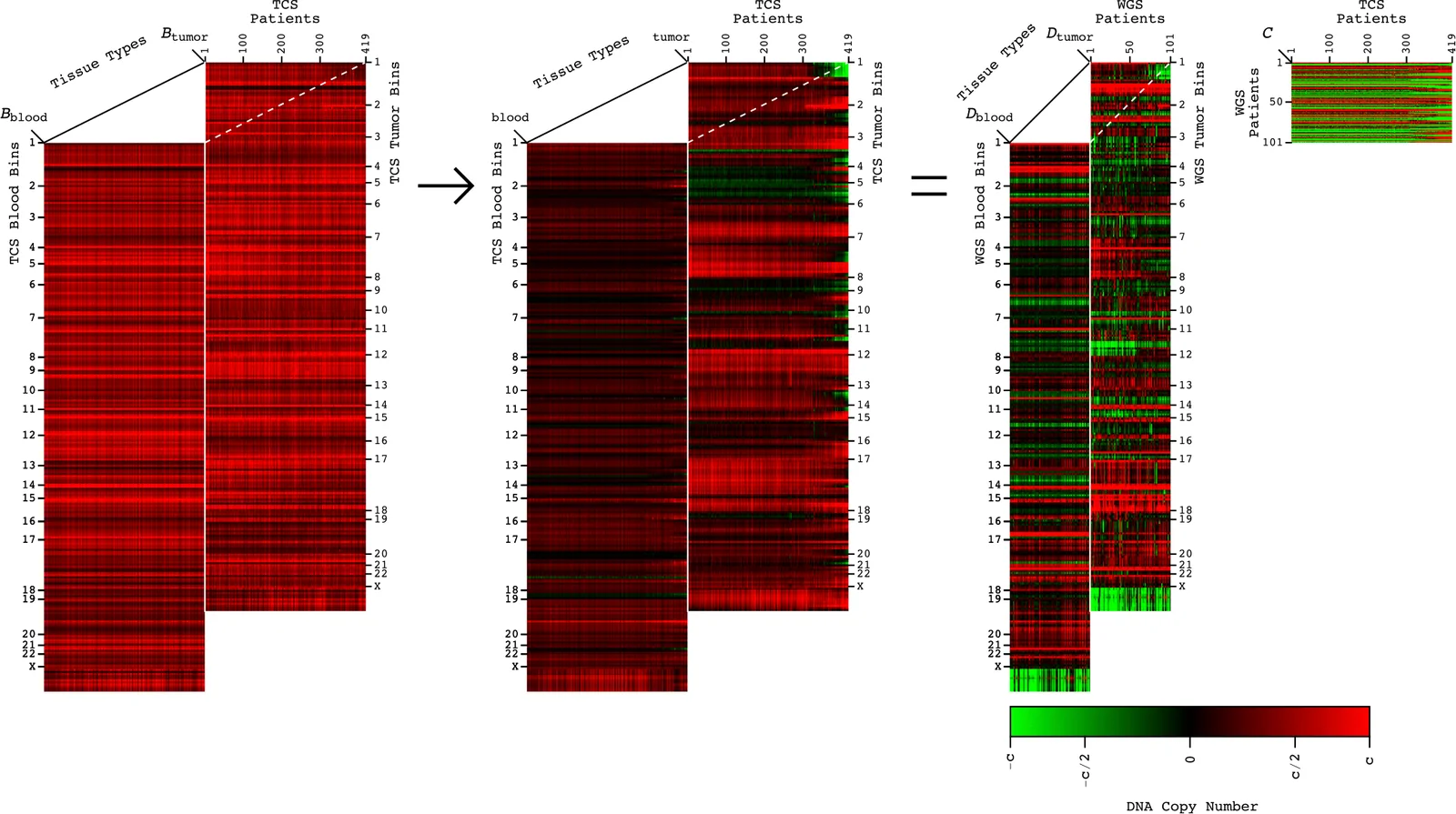
Semisupervised model validation from real-world data at the two-matrix limit. The pseudoinverse projection of Eq. (4) of the Y=419 validation patient-matched tumor and blood TCS profiles onto the X=101 discovery patient-matched genomes spans the $Z_{1}=10\;354$ and $Z_{2}=10\;475$ genomic features shared between the TCS and WGS tumor and blood profiles, respectively. The contrasts of overabundance (red) and underabundance (green) of DNA copy numbers in each TCS profile in the data layers $B_{1}$ and $B_{2}$ and each WGS profile relative to the autosomal median (black) in $D_{1}$ and $D_{2}$ are $c=7.5\times 10^{-4}$ and 1.2, respectively. The contrast of the pseudoinverse projection correlation matrix *C* is 5 × 10^−3^.

**FIG. 7.**
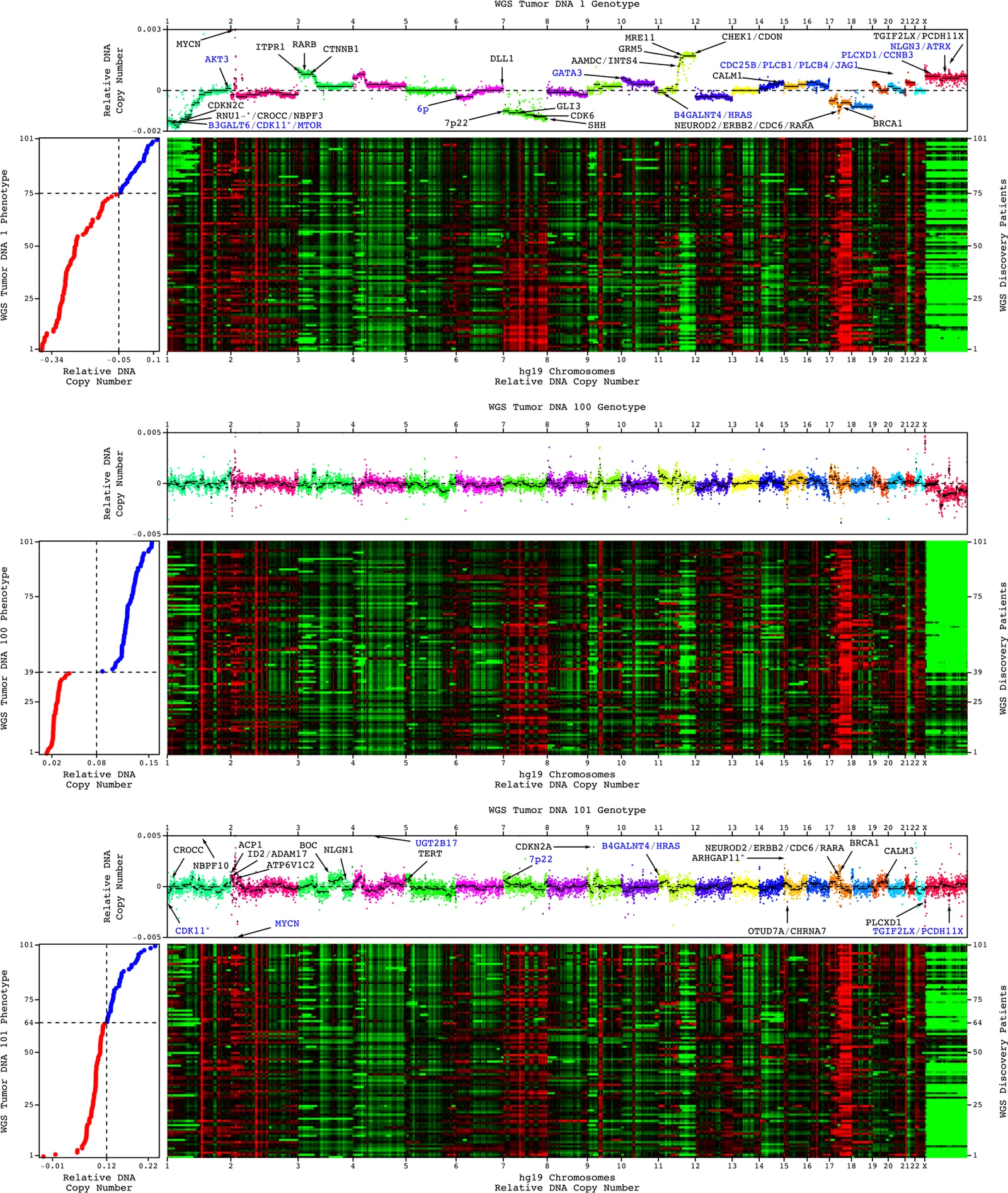
The three GSVD-discovered models of tumor-genotype-to-patient-phenotype relationships. The tumor DNA-specific patterns (i.e., genotypes) $u_{1,k}$, k=1,100,101, are plotted across the $Z_{1}=2,\;831\;960$ tumor genomic features, with NBL-specific (black) and astrocytoma-shared (blue) gene alterations. The patients’ tumor genomes are rasterized, with overabundance (red) and underabundance (green) relative to their autosomal medians (black). The X=101 patients’ tumor samples are sorted and classified into high (blue) and low (red) scores (i.e., phenotypes) in the pattern-derived predictors.

**FIG. 8.**
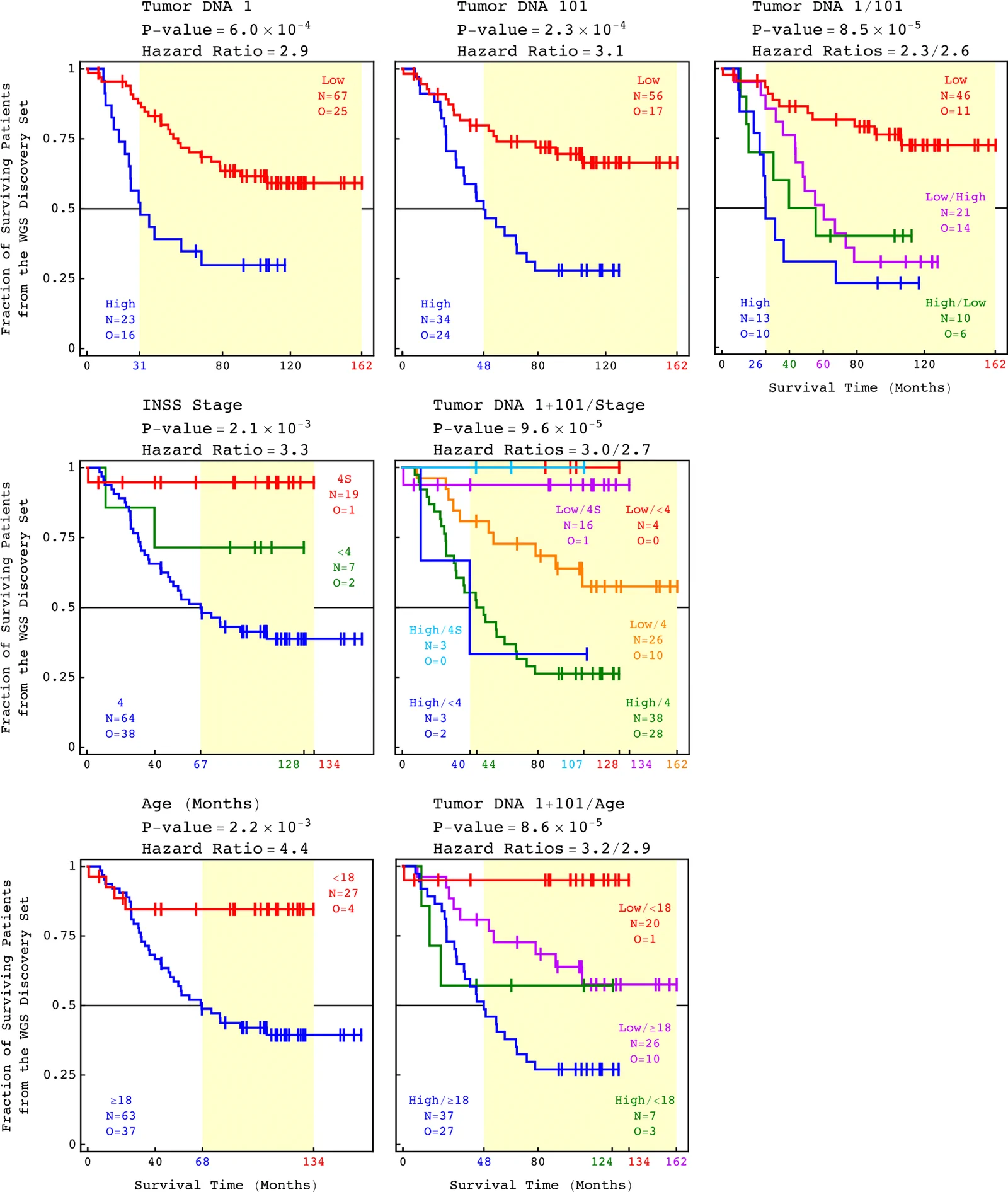
The two GSVD-discovered tumor genomic-specific pattern-derived predictors of NBL patient survival are statistically better than and independent of the standard-of-care indicators INSS stage and age. In the classifications of the 90 of the 101 discovery patients with all labels available, stage and age correspond to KM median survival time differences that are lower and log-rank *P*-values that are greater than the predictors derived from $u_{1,1}$ and $u_{1,101}$ across the 2 831 960 tumor DNA features.

**FIG. 9.**
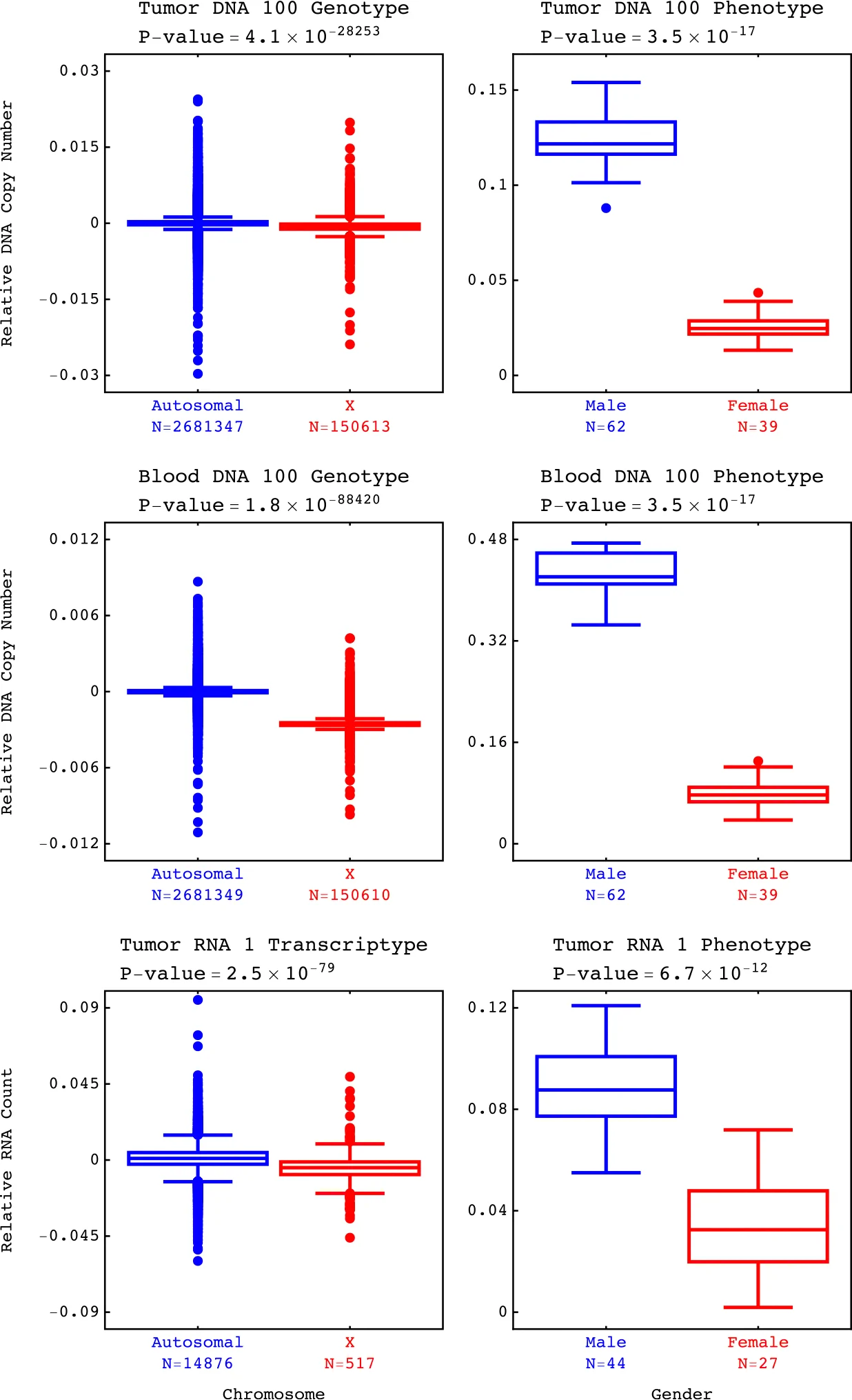
The GSVD genotype- and HO GSVD transcriptype-to-patient-gender-phenotype relationships. The GSVD tumor and blood DNA-specific patterns $u_{1,100}$ and $u_{2,100}$ are of fewer DNA copies, and the HO GSVD RNA pattern $u_{3,1}$ is of fewer RNA counts of X chromosome-mapped features relative to the autosome in the male compared to the female patients.

**FIG. 10.**
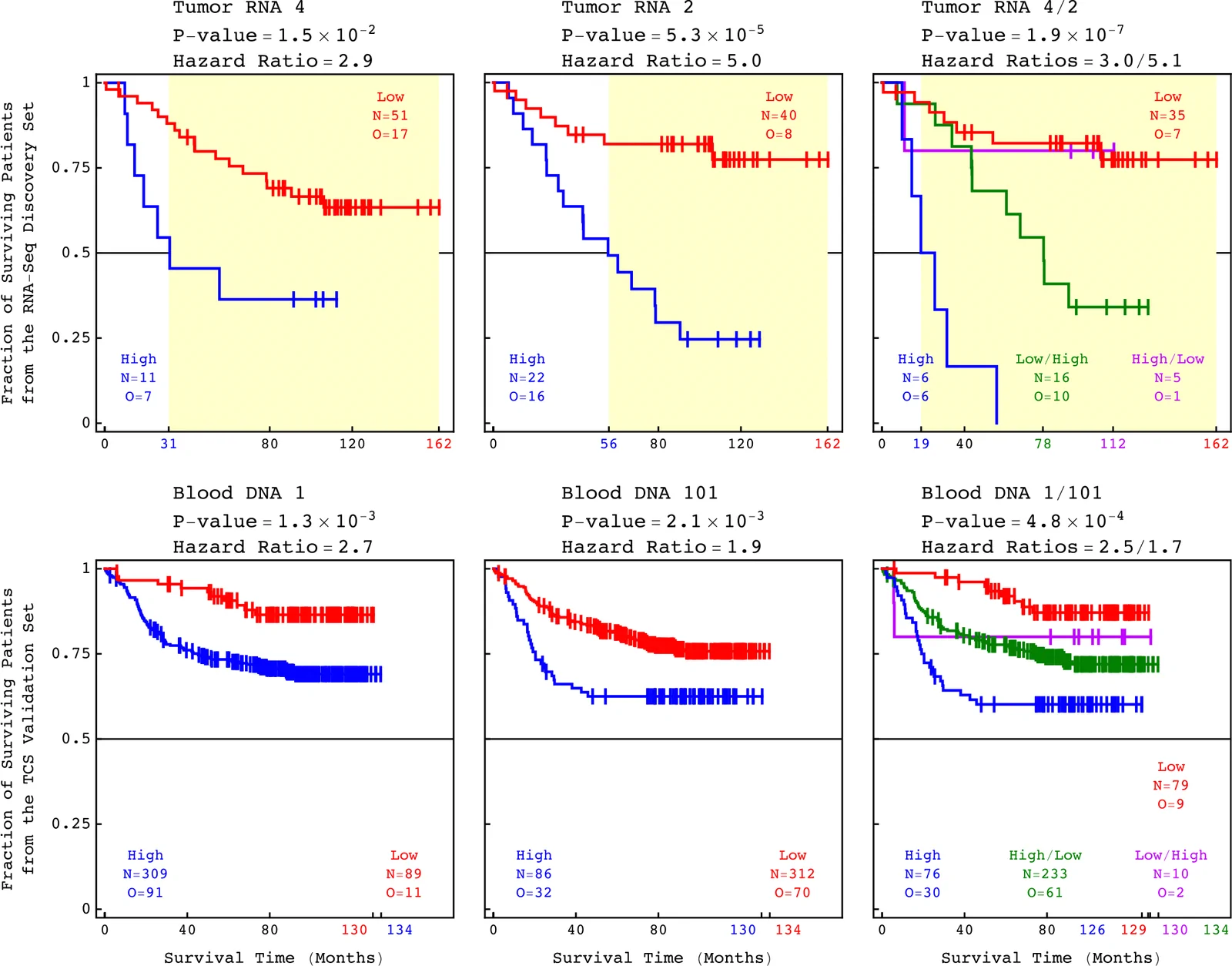
The multiomic representations of the two predictors—GSVD-discovered, HO GSVD-rediscovered, and pseudoinverse projection-validated—are entangled. In the classifications of the 62 patients by the HO GSVD predictors $u_{3,4}$ and $u_{3,2}$ across the 15 393 tumor RNA features, and the 398 validation patients—based upon their TCS profiles, after pseudoinverse projection onto the 101 patients’ WGS profiles—by the GSVD predictors $u_{1,1}$ and $u_{1,101}$ across the 10 475 blood DNA features that are shared between the TCS and WGS profiles, the hazard ratios are consistently within the 95% confidence intervals of those of the GSVD predictors across the 2 831 960 WGS tumor DNA features and among the 90 discovery patients.

**FIG. 11.**
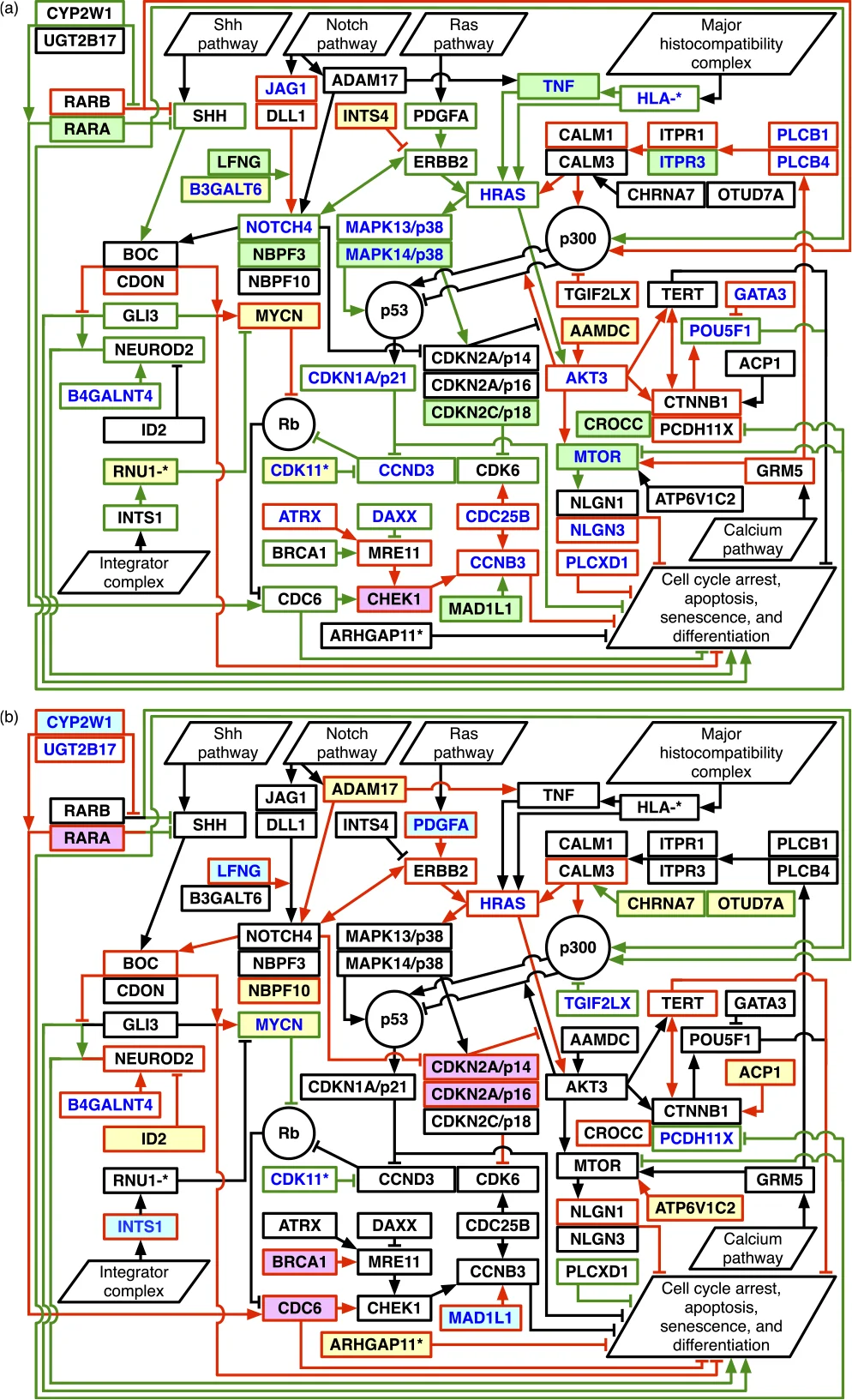
The two predictors—GSVD-discovered, HO GSVD-rediscovered, and pseudoinverse projection-validated—are interpretable in terms of known and new disease mechanisms and drug targets. (a) The predictor that is represented by the GSVD tumor and blood patterns $u_{n,1}$, n=1,2, of the mathematically most tumor-exclusive triplet is depicted in a network of amplified (red) and overexpressed (light red), unaltered (black), or deleted (green) and underexpressed (light green) genes that increase (arrows) or decrease (bars) downstream activities, some of which are independently correlated with survival in NBL (yellow), GBM (blue), or astrocytoma (light blue). (b) The predictor that is represented by $u_{n,101}$, n=1,2, of the tumor- and blood-common triplet.

**FIG. 12.**
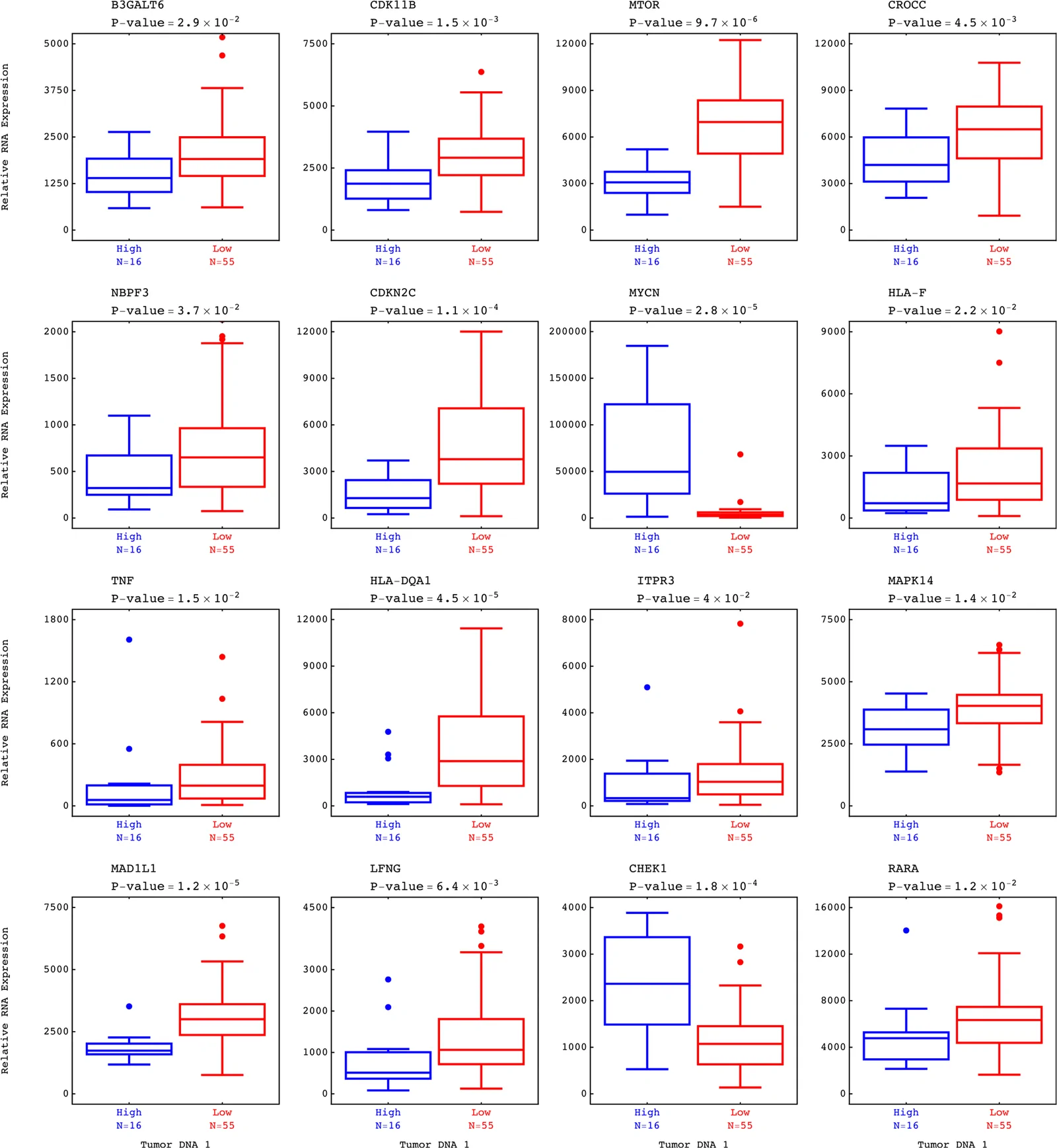
The interpretations of a predictor across its entangled tumor DNA and RNA representations coherently complement each other. Tumor differential RNA expression is consistent with the DNA CNAs in the GSVD $u_{1,1}$-derived predictor.

**FIG. 13.**
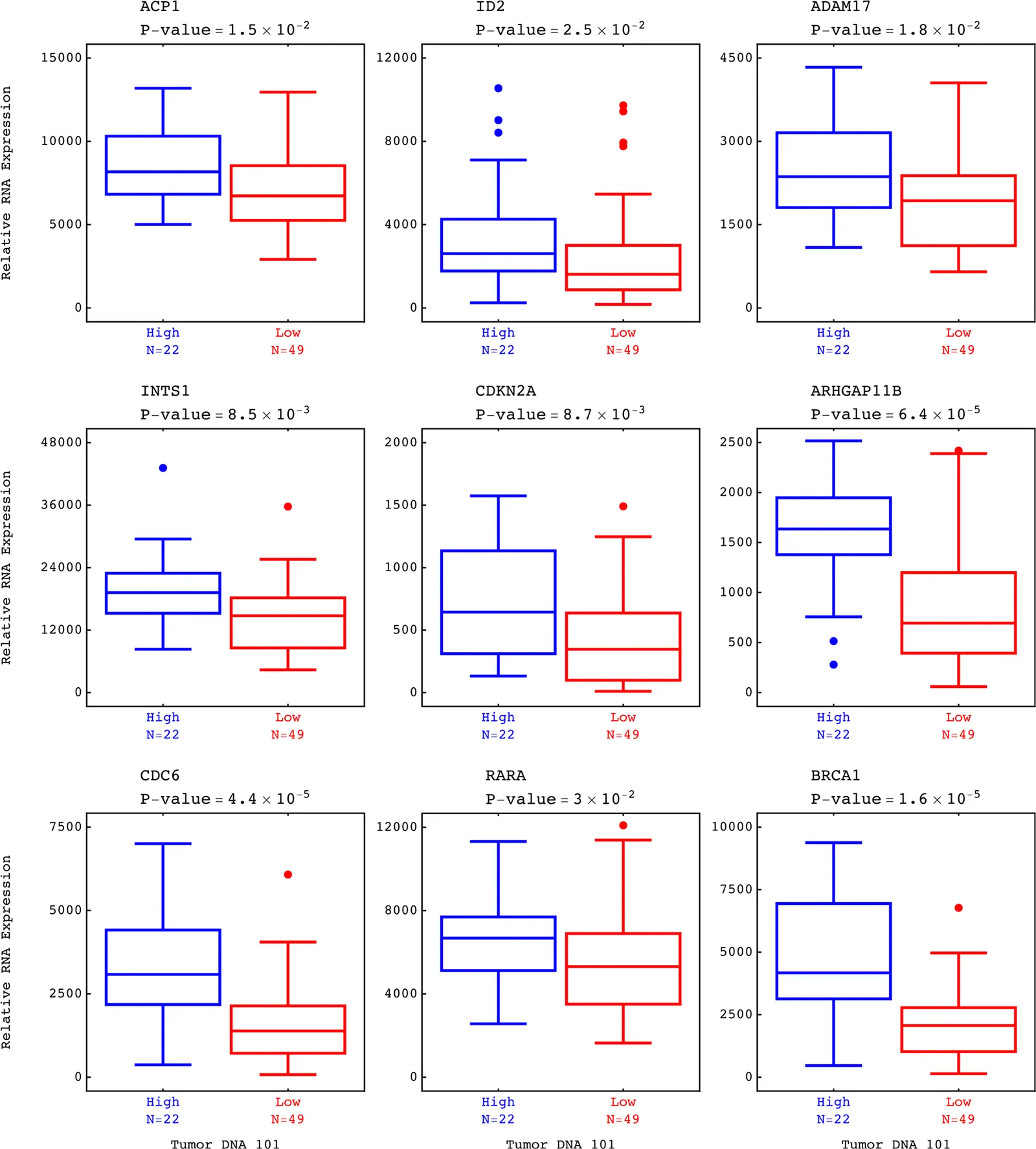
The interpretations of a predictor across its entangled representations coherently complement each other. Tumor differential RNA expression is consistent with the DNA CNAs in the GSVD $u_{1,101}$-derived predictor.

We illustrate the framework in the discovery (Figs. 4 and 5), validation (Fig. 6), and interpretation (Fig. 7) of two novel predictors in neuroblastoma (NBL)—each with three entangled representations—in the tumor and blood genomes and the tumor transcriptome. We show that entanglement holds: the result of the measurement of any one representation approximately determines the results of the measurements of the remaining two, and the prediction of patients’ survival and treatment responses via the measurement of any one representation is consistent with the measurements of the remaining two (Figs. 8–10 and Tables I–III). In every representation, the two predictors combined are consistently more accurate than the best standard-of-care biomarker (i.e., tumor *MYCN* amplification). In addition, the interpretation of any one representation, in terms of known and new disease mechanisms and drug targets, coherently complements the interpretations of the remaining two (Figs. 11–13).

We conclude that our quantum mechanics-based multitensor AI/ML is uniquely able to discover, validate, and interpret predictors from small-cohort, noisy, and high-dimensional multiomic data.

### THE UNIFIED FRAMEWORK: THE MULTITENSOR COMPARATIVE SPECTRAL DECOMPOSITION

Consider a multitensor of N real data layers ${𝒟}_{n}$, n=1,…,N. Each layer ${𝒟}_{n}\in R^{Z_{n}\times X_{2}\times \cdots \times X_{M}}$ is a tensor (i.e., a multiway array) with M dimensions (i.e., axes). The row-axis $z_{n}$ is specific to the n-th layer and corresponds to its specific features (e.g., tumor or blood DNA or tumor RNA sequences). The column-axes $x_{m}$, m=2,…,M, are one-to-one matched across all layers and correspond to the matched samples (e.g., patients or platforms).

We introduce the multitensor comparative spectral decomposition to simultaneously factor the data layers, each into a structure-preserving superposition (i.e., a weighted sum) of rank-one “subtensors” (Fig. 1). The $\left(k,\ell_{2},\ldots ,\ell_{M}\right)$-th subtensor in the n-th layer ${𝒯}_{n,k\ell_{2}\ldots \ell_{M}}\in R^{Z_{n}\times X_{2}\times \cdots \times X_{M}}$, k=1,…,K and $\ell_{m}=1,\ldots ,L_{m}$, is an outer product of one unit vector (i.e., a normalized discretized pattern) across each axis, one layer-specific vector across the independent row-axis $u_{n,k}\in R^{Z_{n}}$, and M-1 vectors across the shared column-axes $v_{\ell_{j}}\in R^{X_{m}}$ that are identical in all layers,

(1) $${𝒯}_{n,k\ell_{2}\ldots \ell_{M}}=u_{n,k}⊗v_{\ell_{2}}⊗\cdots ⊗v_{\ell_{M}},$$

where $\left\|u_{n,k}\right\|=1$, $\left\|v_{\ell_{m}}\right\|=1$, and ⊗ denotes the outer product. Each layer is then expressed as

(2) $$\begin{array}{l} {𝒟}_{n}=\sum_{k=1}^{K}\sum_{\ell_{2}=1}^{L_{2}}\ldots \sum_{\ell_{M}=1}^{L_{M}}r_{n,k\ell_{2}\ldots \ell_{M}}{𝒯}_{n,k\ell_{2}\ldots \ell_{M}}\\ \;={ℛ}_{n}\times_{1}U_{n}\times_{2}V_{2}\times_{3}\cdots \times_{M}V_{M}, \end{array}$$

where $\times_{m}$ denotes the mode-m product (i.e., a tensor-matrix product that contracts the m-th axis of a tensor by applying a matrix along the axis). Notationally, $U_{n}\in R^{Z_{n}\times K}$ is the n-th layer-specific row-axis factor matrix whose k-th column is $u_{n,k}$. Similarly, $V_{m}\in R^{X_{m}\times L_{m}}$ is the m-th shared column-axis factor matrix whose $\ell_{m}$-th column is $v_{\ell_{m}}$. The core tensor ${ℛ}_{n}\in R^{K\times L_{2}\times \cdots \times L_{M}}$ contains the superposition coefficient $r_{n,k\ell_{2}\ldots \ell_{M}}$ (i.e., the weight) of each subtensor in the n-th layer.

To discover a model, each of the M-1 shared factor matrices and, separately, all of the N layer-specific matrices are computed by simultaneously using all samples from all layers of the discovery data and via unsupervised computations that are exact and extend as closely as possible the existence and uniqueness properties of the SVD^38–40^ (Figs. 2, 4, and 5). At the one-matrix limit (i.e., N=1 and M=2), this computation reduces to the SVD of $D\in R^{Z\times X}$,

(3) $$\begin{array}{c} T_{k}=u_{k}⊗v_{k},\\ D=\sum_{k=1}^{K}\sigma_{k}T_{k}=\Sigma \times_{1}U\times_{2}V=U\Sigma V^{T}, \end{array}$$

where the row- and column-axis matrices $U\in R^{Z\times K}$ and $V\in R^{X\times K}$ are column-wise orthonormal, and the core matrix $\Sigma =diag\left(\sigma_{k}\right)\in R^{K\times K}$ is diagonal and positive. The SVD always exists and is always unique up to phase factors of ±1 for each pair of corresponding vectors $u_{k}$ and $v_{k}$ across the row- and column-axes, respectively. This is except in degenerate subspaces defined by subsets of equal singular values $\sigma_{k}$, where the subsets of pairs of corresponding $u_{k}$ and $v_{k}$ are not uniquely defined.

To validate a model-derived predictor, each layer in the validation data is reconstructed in terms of the discovery factor matrices via semisupervised computations that are exact (even if the reconstruction of the validation data is lossy) and extend as closely as possible the properties of the SVD. At the one-matrix limit, this reduces to projecting the validation data $B\in R^{Z\times Y}$ onto the discovery data D via its SVD-computed pseudoinverse^49^ $D^{†}=V\Sigma^{-1}U^{T}$ (Figs. 2 and 6),

(4) $$B\to DC=DD^{†}B=UU^{T}B.$$

The unified framework uniquely solves the challenges of small-cohort, noisy, and high-dimensional data, because the multitensor comparative spectral decomposition always converges into a model, the model is almost always unique, and its computation is lossless (i.e., exact and structure-preserving). These properties mean that the model is also interpretable. The variables of the decomposition of discovery data—the factor matrices, the vectors that form the matrices, and the patterns that the vectors discretize—are interpretable in terms of the underlying interrelated phenomena. Operations with these variables—including reconstruction of validation data in the subspaces that the patterns span—are interpretable in terms of simulated observations of the phenomena and their interrelations.

Moreover, the unified framework offers a cost-effective means to model interrelated phenomena from data, because optimized subroutines exist to compute the SVD and pseudoinverse,^43^ while perturbation theories exist to assess the stability of the computations.

Finally, the fact that the unified framework is multilinear and sometimes orthogonal does not imply that the interrelated phenomena underlying the data are linear and orthogonal. Dynamical systems, chaotic and nonlinear as well as linear ones, in physics and engineering,^44,45^ and now also in biology and medicine,^46–48^ are regularly studied with linear orthogonal transforms. For example, previous SVD modeling uncovered such transforms, including the Fourier transform and its Gaussian analog—the Hermite transform—in transcriptomic profiles from cell time-course and electrophoresis migration-length experiments, respectively.^36,37^

### THE TENSOR HO GSVD: UNSUPERVISED SOLUTION AT THE MULTITENSOR LIMIT AND ITS PROPERTIES

Consider a multitensor of N real-valued M-dimensional tensor data layers, each with all-full column rank (i.e., for all n, unfolding the n-th tensor while preserving its specific axis gives the full column rank matrix $D_{n}\in R^{Z_{n}\times X_{2}\ldots X_{m}}=R^{Z_{n}\times K}$, and for all n and m, unfolding while preserving the shared m-th axis gives the full column rank $D_{n,m}\in R^{Z_{n,m}\times L_{m}}$). The unsupervised solution to the multitensor comparative spectral decomposition of Eqs. (1) and (2) is then the tensor HO GSVD (Fig. 4).

We prove properties of the tensor HO GSVD that are necessary to derive and test a model. Its computation—via the lossless multimatrix HO GSVD^22,23^—is, by definition, exact and structure-preserving.

#### Theorem 1 (Existence).

The tensor HO GSVD of N real M-dimensional all-full column rank tensors exists.

##### Proof.

Each of the M-1 shared factor matrices $V_{m}$ is computed from the HO GSVD of the N tensors ${𝒟}_{n}$ unfolded into the corresponding full column rank matrices $D_{n,m}$,

(5) $$\begin{array}{c} T_{n,\ell_{m}}=u_{n,\ell_{m}}⊗v_{\ell_{m}},\\ D_{n,m}=\sum_{\ell_{m}=1}^{L_{m}}\sigma_{n,\ell_{m}}T_{n,\ell_{m}}=\Sigma_{n,m}\times_{1}U_{n,m}\times_{2}V_{m}\\ =U_{n,m}\Sigma_{n,m}V_{m}^{T}. \end{array}$$

From the existence of the HO GSVD of the N matrices $D_{n,m}$, there exists an invertible and column-wise normalized $V_{m}\in R^{L_{m}\times L_{m}}$ for all m.

Similarly, all the N tensor-specific matrices $U_{n}$ are computed from the HO GSVD of all the tensors unfolded into the full column rank matrices $D_{n}$,

(6) $$\begin{array}{c} T_{n,k}=u_{n,k}⊗v_{k},\\ D_{n}=\sum_{k=1}^{K}\sigma_{n,k}T_{n,k}=\Sigma_{n}\times_{1}U_{n}\times_{2}V=U_{n}\Sigma_{n}V^{T}. \end{array}$$

From the existence of the HO GSVD of the N matrices $D_{n}$, there exists a column-wise normalized tensor-specific matrix $U_{n}\in R^{Z_{n}\times K}$ for all n.

Given $U_{n}$ and $V_{m}$ for all n and m, each core tensor ${ℛ}_{n}\in R^{K\times L_{2}\times \cdots \times L_{M}}$ is computed from the corresponding matrix $D_{n}$, where from Eqs. (1), (2), (5), and (6),

(7) $$\begin{array}{l} D_{n}=U_{n}R_{n}\left(V_{2}^{T}⊗\cdots ⊗V_{M}^{T}\right),\\ R_{n}=\Sigma_{n}\left(V_{2}^{-T}⊗\cdots ⊗V_{M}^{-T}\right), \end{array}$$

and where ⊗ denotes the Kronecker product. From the existence of the HO GSVD of the N matrices $D_{n}$, there also exists $R_{n}\in R^{K\times L}$, $L=L_{2}\ldots L_{M}$, which is foldable into ${ℛ}_{n}$.

Consequently, the tensor HO GSVD of N real M-dimensional, all-full column rank tensors exists. □

In the HO GSVD of the tensors ${𝒟}_{n}$ unfolded into the full column rank matrices $D_{n}$, the shared matrix V is formed from the eigenvectors of the arithmetic mean S of all pairwise quotients $A_{i}A_{j}^{-1}$ of the matrices $A_{i}=D_{i}^{T}D_{i}$,

(8) $$\begin{array}{c} S=\frac{1}{N(N\;-\;1)}\sum_{i=1}^{N}\sum_{j>i}^{N}\left(A_{i}A_{j}^{-1}+A_{j}A_{i}^{-1}\right),\\ SV=V\Lambda , \end{array}$$

where $S\in R^{K\times K}$ and $\Lambda =diag\left(\lambda_{k}\right)\in R^{K\times K}$ is diagonal and positive.^22^

We proved that S has a real eigensystem, with K independent eigenvectors $v_{k}$, such that the inverse of V exists.^23^ Each tensor-specific factor matrix $U_{n}$ is then computed by projecting the corresponding $D_{n}$ onto $V^{-T}$. Consequently, the N matrices $U_{n}$ are unique, up to phase factors of ±1 of each set of vectors $u_{n,k}$ for a given k, except in degenerate subspaces defined by subsets of equal eigenvalues $\lambda_{k}$. Similarly, each of the M shared matrices $V_{m}$ is unique, up to phase factors of ±1 of each set of vectors $v_{\ell_{m}}$, except in degenerate subspaces defined by subsets of equal eigenvalues $\lambda_{\ell_{m}}$.

#### Theorem 2 (Uniqueness properties).

The tensor HO GSVD of N real M-dimensional all-full column rank tensors is unique, except in degenerate subspaces defined by subsets of equal eigenvalues (i.e., $\lambda_{k}$ or $\lambda_{\ell_{m}}$), and up to a phase factor of ±1 of each set of N tensor-specific vectors $u_{n,k}$ for a given k and for all n, and all their corresponding coefficients $r_{n,k\ell_{2}\ldots \ell_{M}}$ for the given k and for all n and $\ell_{m}$. Similarly, the uniqueness is also up to a phase factor of ±1 of each shared vector $v_{\ell_{m}}$ and all of its corresponding coefficients.

##### Proof.

Similar to the proven uniqueness properties of the GSVD and tensor GSVD,^30,53^ the uniqueness properties of the tensor HO GSVD follow from those of the factor matrices $U_{n}$ and $V_{m}$ for all n and m. □

Several special cases of the tensor HO GSVD follow.

##### Corollary 1 (HO GSVD).

The tensor HO GSVD of N real M-dimensional all-full column rank tensors is equivalent to the HO GSVD of the corresponding matrices.

##### Corollary 2 (Tensor GSVD).

The tensor HO GSVD of N real M-dimensional all-full column rank tensors, N-1 of which are identical (i.e., ${𝒟}_{n}={𝒟}_{1}$, n=1,…,N-1), reduces to the tensor GSVD of ${𝒟}_{1}$ and ${𝒟}_{N}$.

##### Corollary 3 (Tensor SVD).

The tensor HO GSVD of N real M-dimensional all-full column rank tensors, where N-1 are identical (i.e., ${𝒟}_{n}={𝒟}_{1}$, n=1,…,N-1) and where unfolding the N-th tensor while preserving the tensor-specific axis gives the identity matrix (i.e., $D_{N}=I\in R^{K\times K}$*), reduces to the tensor SVD of* ${𝒟}_{1}$.

### MULTITENSOR METRICS TO DERIVE, TEST, AND EXPLAIN A MODEL

We define metrics that are necessary for the mathematical properties of a multitensor comparative spectral decomposition model to be consistent with its interpretation in terms of the interrelated phenomena that underlie the data.

#### Multitensor joint fraction:

Generalizing the SVD fraction,^36,45^ the multitensor joint fraction measures the joint weight of the $\left(k,\ell_{2},\ldots ,\ell_{M}\right)$-th set of N subtensors $w_{k\ell_{2}\ldots \ell_{M}}=\sum_{n=1}^{N}r_{n,k\ell_{2}\ldots \ell_{M}}^{2}$ relative to the total weight of the data,

(9) $$f_{k\ell_{2}\ldots \ell_{M}}=\frac{w_{k\ell_{2}\ldots \ell_{M}}}{\sum_{k=1}^{K}\sum_{\ell_{2}=1}^{L_{2}}\cdots \sum_{\ell_{M}=1}^{L_{M}}w_{k\ell_{2}\ldots \ell_{M}}},$$

such that $0\leq f_{k\ell_{2}\ldots \ell_{M}}\leq 1$. When all subtensor sets have equal weight in the data, $f_{k\ell_{2}\ldots \ell_{M}}=1/\left(KL_{2}\ldots L_{M}\right)$ for all k and $\ell_{m}$. When $f_{k\ell_{2}\ldots \ell_{M}}>1/\left(KL_{2}\ldots L_{M}\right)$, the $\left(k,\ell_{2},\ldots ,\ell_{M}\right)$-th subtensors and the vectors $u_{n,k}$ and $v_{\ell_{m}}$ that form them are mathematically non-negligible.^40–42^ For consistency, the patterns that these vectors discretize are interpreted in terms of interrelated phenomena present in the data.

#### Multitensor joint Shannon entropy:

Generalizing the SVD Shannon entropy,^36,45^ the multitensor joint Shannon entropy measures the complexity of the data in terms of the distribution of the joint fractions over all sets of $\left(k,\ell_{2},\ldots ,\ell_{M}\right)$-th subtensors,

(10) $$e=-\frac{\sum_{k=1}^{K}\sum_{\ell_{2}=1}^{L_{2}}\cdots \sum_{\ell_{M}=1}^{L_{M}}f_{k\ell_{2}\ldots \ell_{M}}\;ln\;(f_{k\ell_{2}\ldots \ell_{M}})}{ln\;\left(KL_{2}\ldots L_{M}\right)},$$

such that 0≤e≤1. An entropy of e=0 corresponds to ordered and redundant data, whose total weight is captured by one subtensor set of given k and $\ell_{m}$. An entropy of e=1 corresponds to disordered and random data, where all sets of subtensors are of equal weight.

#### Multitensor comparative angular distance:

Extending the GSVD and tensor GSVD angular distances,^16,29^ the multitensor comparative angular distance measures the pairwise-commonality of two subtensors from the $\left(k,\ell_{2},\ldots ,\ell_{M}\right)$-th set in terms of their relative weights in the corresponding data layers ${𝒟}_{i}$ and ${𝒟}_{j}$,

(11) $$\theta_{ij,k\ell_{2}\ldots \ell_{M}}=arctan\left|\frac{r_{i,k\ell_{2}\ldots \ell_{M}}}{r_{j,k\ell_{2}\ldots \ell_{M}}}\right|-\pi /4,$$

where $-\pi /4\leq \theta_{ij,k\ell_{2}\ldots \ell_{M}}\leq \pi /4$. When $\theta_{ij,k\ell_{2}\ldots \ell_{M}}=0$, the two subtensors, and the vectors that form them, are mathematically of equal weight. For consistency, the corresponding patterns are interpreted to represent phenomena common to both layers. At the limits of $\theta_{ij,k\ell_{2}\ldots \ell_{M}}=\pm \pi /4$, the subtensor in the i-th layer is mathematically either of maximum or minimum weight relative to that in the j-th layer, and the patterns are interpreted to represent phenomena exclusive to either the i-th or the j-th layer, respectively.

From Eq. (7), it follows that the tensor HO GSVD angular distance equals the HO GSVD angular distance of the two subtensors unfolded into submatrices from the (k,ℓ)-th set and in terms of their relative weights in the two data layers unfolded into matrices $D_{i}$ and $D_{j}$, where all unfoldings preserve the layer-specific axes,

(12) $$\theta_{ij,k\ell_{2}\ldots \ell_{M}}=\theta_{ij,k}=arctan\left(\frac{\sigma_{i,k}}{\sigma_{j,k}}\right)-\pi /4.$$

Previously, we additionally defined the inverse eigenvalue as a measure of the all-commonality of the (k,ℓ)-th set of submatrices in the set of layers $D_{n}$.^23^ We proved that the inverse of the eigenvalue $\lambda_{k}$ of Eq. (8) satisfies

(13) $$0<\lambda_{k}^{-1}\leq 1,$$

where $\lambda_{k}^{-1}=1$ if and only if the corresponding angular distances of Eqs. (10) and (11) satisfy $\theta_{ij,k}=0$ for all pairs of i≠j. In this case, the HO GSVD maintains partial or, like the GSVD, even full column-wise orthogonality of $U_{n}$.^21^ For consistency, the patterns that the all-common vectors discretize are interpreted to represent phenomena common to all layers.

### NOVEL CONNECTION TO THE QUANTUM MECHANICAL CONCEPT OF “ENTANGLEMENT”

Entanglement and superposition emerge from the wave-particle duality concept as the two fundamental principles at the mathematical foundation of quantum mechanics.^62^

Superposition allows a quantum system to simultaneously exist in a set of states—leading to a probability distribution for the result of a quantum measurement of the system. Entanglement allows each state to be linked with other states of other systems, such that the pre- and post-measurement probabilities are given by a joint distribution of all the linked systems, and the result of a measurement of any one system determines the results of the measurements of all the other ones.

We highlight the novel connection of the unified framework to the quantum mechanical concept of entanglement in addition to that of superposition (Fig. 3). The $\left(x_{2}\ldots x_{M}\right)$-th set of N one-to-one mapped samples $d_{n,x_{2}\ldots x_{M}}={\left|d_{n}\right\rangle }_{x_{2}\ldots x_{M}}\in R^{Z_{n}}$, one per data layer ${𝒟}_{n}$ and across its specific features, corresponds to a set of entangled systems ${\left|d_{1}\right\rangle }_{x_{2}\ldots x_{M}}⊗\cdots ⊗{\left|d_{N}\right\rangle }_{x_{2}\ldots x_{M}}=\prod_{n=1}^{N}{\left|d_{n}\right\rangle }_{x_{2}\ldots x_{M}}$, where $\prod_{n=1}^{N}$ represents a series of N outer products, and where entanglement is consistent with each data layer measuring multiple aspects of interrelated phenomena.

The unified framework rewrites the ($x_{2}\ldots x_{M}$)-th entangled sample set as a superposition of states, where each state is represented by a set of entangled vectors $u_{n,k}={\left|u_{n}\right\rangle }_{k}$ or discretized patterns, one per data layer,

(14) $$\prod_{n=1}^{N}{\left|d_{n}\right\rangle }_{x_{2}\ldots x_{M}}=\left(v_{\ell_{2},x_{2}}\ldots v_{\ell_{M},x_{M}}\right)\sum_{k=1}^{K}\prod_{n=1}^{N}r_{n,k\ell_{2}\ldots \ell_{M}}{\left|u_{n}\right\rangle }_{k}.$$

Without loss of generality, let the layer-specific factor matrix $U_{N}$ be column-wise orthonormal, such that $K{\left\langle u_{N}|u_{N}\right\rangle }_{k}=\delta_{Kk}$, where $\delta_{Kk}$ is the Dirac delta function. A measurement of the N-th data layer that results in ${\left|u_{N}\right\rangle }_{K}$ then determines the results of the measurements of all the other layers,

(15) $$\begin{array}{ll} {\left|u_{N}\right\rangle }_{KK}\left\langle u_{N}\right|\prod_{n=1}^{N}{\left|d_{n}\right\rangle }_{x_{2}\ldots x_{M}}= & \left(v_{\ell_{2},x_{2}}\ldots v_{\ell_{M},x_{M}}\right)\\ \; & \;\times \prod_{n=1}^{N-1}r_{n,K\ell_{2}\ldots \ell_{M}}{\left|u_{n}\right\rangle }_{K}{\left|u_{N}\right\rangle }_{K}. \end{array}$$

In this context, the multitensor joint fraction of Eq. (9) provides the joint probability for the measurement of each set of one-to-one mapped samples to result in any one set of entangled patterns representing the same state. The multitensor joint Shannon entropy of Eq. (10), then, quantifies the complexity of the data in terms of the joint probability distribution over all possible measurement outcomes, that is, over all possible states or sets of entangled patterns.

### NOVEL PREDICTORS WITH ENTANGLED MULTIOMIC REPRESENTATIONS IN NBL PATIENTS’ TUMOR AND BLOOD GENOMES AND TUMOR TRANSCRIPTOMES

We illustrate the unified framework in the modeling of multiomic data from NBL patients.^63,64^ Prediction, together with understanding and management, of the outcomes of this rare disease, which range from spontaneous regression to relapse and death, remain limited, and rely mostly on stage, age, and *MYCN* status, none of which are NBL-specific.^65^

### GSVD DISCOVERY OF PREDICTORS IN NBL PATIENTS’ TUMOR GENOMES

We use the unsupervised multitensor comparative spectral decomposition at the two-matrix limit (i.e., the GSVD) to model minimally preprocessed tumor and blood genomes from the Therapeutically Applicable Research to Generate Effective Treatments (TARGET) project^66^ (Mathematica Notebook 1 and Dataset 1). The tumor and blood DNA data layers $D_{1}$ and $D_{2}$ are of X=101 matched columns, corresponding to the patient samples, and $Z_{1}=2\;831\;960$ and $Z_{2}=2\;831\;959$ independent rows, corresponding to the genomic features (i.e., nonoverlapping 1K-nucleotide bins across the autosome and the X chromosome of the hg19 reference human genome^67^) (Fig. 5). The layers tabulate the log_2_ of the Complete Genomics WGS read counts, with each profile centered at its autosomal median.^68^

The GSVD separates the tumor and blood data layers into K=101 triplets of patterns. Each triplet includes one shared pattern across the patient samples $v_{k}^{T}$, one tumor layer-specific pattern across the $Z_{1}$ tumor genomic features $u_{1,k}$, and one blood-specific pattern across the $Z_{2}$ blood features $u_{2,k}$. Like previous GSVD, HO GSVD, and tensor GSVD models, this model is robust to (i.e., quantitatively similar and qualitatively the same under) perturbations to the data (e.g., changing the bin sizes in the range of 100–2.5K nucleotides).

Eight triplets are of multitensor joint fractions of $f_{k}≳3/K$ (out of 22 triplets of $f_{k}>1/K$), as is reflected by the multitensor joint Shannon entropy of e=0.77. Among these, one triplet is mathematically the most exclusive to the tumor genomes, with its GSVD angular distance $\theta_{1}>max\left(\pi /5,\theta_{k}\right)$, k=2,…,101. Two are mathematically with the greatest magnitude in the blood relative to the tumor genomes while also being common to both, with $-\pi /5<\left\{\theta_{101},\theta_{100}\right\}<min\left(-\pi /15,\theta_{k}\right)$, k=1,…,99 (Fig. 7).

Similar to previous GSVD models, the predictor derived from the tumor-specific pattern $u_{1,1}$ in the mathematically most tumor-exclusive triplet is correlated with overall survival. Its scores in the tumor profiles, cut off at one standard deviation from the mean, classify the 90 of the 101 patients with all labels available into two groups, with a 131-month Kaplan-Meier (KM) median survival difference, a 2.9 univariate Cox hazard ratio, and a 0.77 concordance index or accuracy (log-rank and Wald *P*-values = 6.0 × 10^−4^ and 1.0 × 10^−3^) (Fig. 8 and Table I).

Unlike previous GSVD models, the predictor derived from the tumor pattern $u_{1,101}$ in the mathematically most blood-exclusive triplet, which is common to the tumor and blood data layers, is also correlated with survival. It classifies the patients into two groups, with a 114-month survival difference, a 3.1 hazard ratio, and a 0.74 concordance (log-rank and Wald *P*-values = 2.3 × 10^−4^ and 4.5 × 10^−4^). The patterns $u_{1,1}$ and $u_{1,101}$ are mathematically orthogonal to each other^21^ (i.e., uncorrelated). KM survival analyses and Cox proportional hazards models show that the derived predictors are also statistically independent of each other (i.e., with their univariate hazard ratios within the 95% confidence intervals of the bivariate ratios). As a result, the two predictors combined are statistically better than either one alone. For example, their maximum median survival difference of 136 months is greater, and the log-rank *P*-value and Akaike information criterion (AIC) values are lower, than those of either predictor alone.

The two predictors combined are also statistically better than and independent of all standard-of-care indicators, including the International Neuroblastoma Staging System (INSS) stage, age,^69^ MYCN status, histopathology, DNA ploidy, the Children’s Oncology Group (COG) risk, and the mitosis-karyorrhexis index (MKI). The risk that the tumor’s whole genome confers upon survival, as it is reflected by the bivariate ratios of the predictors combined with each standard indicator, is greater than that conferred by any indicator except for histopathology.

Taken together, the two predictors are as clinically actionable as any of the standard indicators.

### GSVD DISCOVERY OF REPRESENTATIONS OF THE PREDICTORS IN THE PATIENTS’ BLOOD GENOMES

The classifications of the patients by the representations of the predictors derived from the GSVD blood DNA-specific patterns $u_{2,1}$ and $u_{2,101}$ are approximately the same as those by their entangled tumor patterns $u_{1,1}$ and $u_{1,101}$ (e.g., with the univariate hazard ratios of the blood patterns within the 95% confidence intervals of those of the tumor patterns) (Table II).

Similar to the tumor patterns, the blood patterns are mathematically orthogonal and statistically independent.

### GSVD BLIND SEPARATION OF THE PREDICTORS FROM NORMAL GENOTYPE-PHENOTYPE VARIATIONS

The GSVD blindly (e.g., label-free and without feature engineering) separates the tumor and blood DNA representations of the two predictors from normal demographic variations among the patients that are present in the blood and are conserved in the tumors. The second most blood-exclusive triplet (i.e., the 100th triplet), which is also common to the tumor and blood genomes, is correlated with the normal X chromosome-number variation between the male and female patient samples. Both 100th tumor- and blood-specific patterns $u_{1,100}$ and $u_{2,100}$ describe, on average, fewer copy numbers of genomic features that map to the X chromosome than to the autosome [Mann–Whitney–Wilcoxon (MWW) *P*-values < 10^−28,252^]. Each of the entangled patterns classifies the patients by gender (hypergeometric and MWW *P*-values = 6.8 × 10^−29^ and 3.5 × 10^−17^) (Fig. 9).

Blind separation of the disease-specific patterns $u_{n,k}$, k=1,101, from normal variations $u_{n,100}$, n=1,2, means that the predictors perform independently of the demographic variations among the patients. For example, the two predictors perform consistently well among the 56 male patients as well as among the 34 female patients (e.g., with their univariate hazard ratios within the 95% confidence interval of those of all the 90 patients).

Blind separation also means that the algorithms perform independently of the demographic variations among the genomes. Most approaches consider features that normally vary among demographies to be confounding and exclude the known ones. For example, the TCGA study of ovarian cystadenocarcinoma (OV) excluded the X chromosome.^70^ Modeling these TCGA data with the GSVD and tensor GSVD, however, we discovered, validated, and interpreted a predictor that is encompassed within the X chromosome.^29,30^ This predictor associates OV patients’ survival and treatment responses with aberrations in X chromosome-mapped genes that are consistent across their tumor DNA copy numbers and RNA and protein expression levels. Similarly, the patterns that represent the two predictors in the NBL patients’ tumor and blood DNA include disease-specific alterations that map to the X chromosome.

### HO GSVD DISCOVERY OF REPRESENTATIONS OF THE PREDICTORS IN THE PATIENTS’ TUMOR TRANSCRIPTOMES

We use the unsupervised multitensor comparative spectral decomposition at the multimatrix limit (i.e., the HO GSVD) to model minimally preprocessed tumor and blood genomes and tumor transcriptomes from the TARGET project. The tumor and blood DNA and tumor RNA data layers $D_{1}$, $D_{2}$, and $D_{3}$ are of X=71 matched columns, corresponding to 71 of the 101 patients with RNA sequencing (RNA-Seq) profiles, and $Z_{1}=2\;831\;960$, $Z_{2}=2\;831\;959$, and $Z_{3}=15\;393$ independent rows, corresponding to the genomic and transcriptomic features. The tumor RNA layer tabulates the log_2_ of the Illumina Hi-Seq 2000-measured RNA read counts.

The HO GSVD separates the tumor and blood genomic and tumor transcriptomic data layers into quartets of patterns, each including one tumor RNA-specific pattern across the $Z_{3}$ tumor RNA features. Four of the K=71 quartets are of inverse eigenvalues $\lambda_{k}^{-1}<8.0\times 10^{-3}$ and carry significant weights in the data. There is a one-to-one correspondence between three of these four HO GSVD quartets and the three GSVD triplets: Predictors derived from the three sets of entangled HO GSVD tumor and blood genomic and tumor transcriptomic patterns classify 62 of the 71 patients with all labels available approximately the same as the GSVD predictors (e.g., with univariate hazard ratios that are within the 95% confidence intervals of each other) (Fig. 10 and Table II).

The first HO GSVD quartet, which carries the greatest weight in the data, corresponds to the 100th GSVD triplet. It classifies the patients by gender (MWW *P*-value < 10^−11^), showing that the HO GSVD, like the GSVD, blindly separates the normal variations from each omic representation of each predictor. The second and fourth HO GSVD quartets correspond to the 101st and first GSVD triplets, respectively. Unlike the GSVD patterns, the HO GSVD patterns are not necessarily mathematically orthogonal. Similar to the two entangled GSVD representations of each of the two predictors, however, the three entangled HO GSVD representations of the two predictors are statistically independent in each of the data layers, including the tumor RNA in addition to the tumor and blood DNA.

### PSEUDOINVERSE PROJECTION CROSS-PLATFORM VALIDATION OF THE REPRESENTATIONS OF THE PREDICTORS IN NBL PATIENTS’ TUMOR AND BLOOD GENOMES

To validate the derived predictors, we use the semisupervised multitensor comparative spectral decomposition (i.e., the pseudoinverse projection) to model tumor and blood DNA validation data in terms of the discovery data. The validation data layers $B_{1}$ and $B_{2}$ are of Y=419 matched columns, corresponding to 419 TARGET patients that are mutually exclusive of (and of different normal demographic variations than) the 101 patients (Dataset 2). The $Z_{1}=10\;354$ and $Z_{2}=10\;475$ independent rows correspond to the genomic features (i.e., tumor and blood Illumina Hi-Seq 2500-measured target-capture sequencing (TCS) bins that are shared with the tumor and blood WGS bins).

The predictors derived from the tumor and blood WGS profiles of the discovery set of 101 patient samples perform consistently well in the TCS profiles of the validation set of 419 patient samples (e.g., with the univariate hazard ratios of either the tumor or blood profiles of one set within the 95% confidence intervals of those of the other) (Figs. 8 and 10 and Tables I–III). The pairs of tumor and blood patterns are not necessarily mathematically orthogonal to each other across the TCS genomic features, unlike how they are across the WGS features. Similar to the WGS data, however, the pairs of representations of the two predictors are statistically independent in the TCS data.

In both the tumor and blood DNA profiles, measured by both the WGS and TCS platforms, and in both the discovery and validation sets of patients, the two predictors combined are at 73%–80% concordance with survival and are consistently more accurate than *MYCN*.

### THE PREDICTORS DESCRIBE ORTHOGONAL MECHANISMS OF PROLIFERATION, CELL DEATH EVASION, AND RESISTANCE TO THERAPY

To interpret the cellular pathways that underlie the two predictors, we determine the status of the circular binary segmentation (CBS)^71^-identified regions in their corresponding representations in the NBL tumor whole genomes $u_{1,k}$, k=1,101. The copy-number mean of an amplified or a deleted segment is at one standard deviation or more from either the autosomal mean or the mean of the chromosome it maps to when the directions of the deviations from the chromosome and the autosome are consistent. These altered segments include most known and as many previously unrecognized DNA CNAs in NBL.

Several segments are correlated with survival independent of the “genetic background” described by the patterns. For example, in the pattern $u_{1,1}$, one amplified segment of 142K nucleotides on chromosome 2 band 2p24.3 encompasses *MYCN*. The amplification of this segment is accompanied by *MYCN* RNA overexpression in the tumor transcriptomes of patients with high scores in the corresponding predictor. The copy numbers of this segment in the tumor genomes classify the patients into two groups, with a 118-month median survival difference, a 2.6 hazard ratio, and a 0.75 concordance index (log-rank and Wald *P*-values = 1.4 × 10^−3^ and 2.0 × 10^−3^). In the pattern $u_{1,101}$, an amplified segment that encompasses the oncogene ID2 is also accompanied by its RNA overexpression and is independently correlated with survival. That these two known oncogene amplifications, which are both recognized as drivers of NBL, map to two statistically independent predictors of survival, which are represented by two mathematically uncorrelated patterns, is in agreement with the orthogonal roles of *MYCN* and *ID2* in promoting cell proliferation^72^ (Figs. 7 and 11, and Dataset 3).

By identifying previously unrecognized alterations that are independently correlated with survival, the two patterns predict new oncogenes to be driving NBL. For example, the amplifications of *AAMDC* in $u_{1,1}$, and of *ADAM17* and a region overlapping *NBPF10* in $u_{1,101}$, are each independently correlated with shorter median survival times. The *NBPF10* gene is a member of the human-specific NBL breakpoint family, so-called because another member, *NBPF1*, was discovered in a screen for genes disrupted by a somatic translocation in an NBL patient. The prediction that each of these genes functions as a driver of NBL is additionally supported by existing evidence for the orthogonal roles of *AAMDC* and *ADAM17* in evading apoptosis^73,74^ (i.e., programmed cell death) and for the association of *NBPF10* with the human-specific brain-size determinants *NOTCH2NLA/B*.^75^

By determining alterations in the NBL predictors that consistently overlap with DNA CNAs in astrocytoma tumors, the patterns also predict new mechanisms underlying the disease, for example, orthogonal mechanisms for the aneuploid tumor cells to evade cell death. In the NBL pattern $u_{1,1}$, and in the brain tumors, this is accomplished via the deletion of the apoptosis-inducing tumor suppressor *TNF* together with the deletion and amplification of *POU5F1* (also known as *OCT4*) and *GATA3*, respectively. *POU5F1* overexpression and *GATA3* underexpression are characteristic of cells in the extra-embryonic tissue, where aneuploid cells are maintained during embryonic development.^76^ In the NBL pattern $u_{1,101}$, evasion of cell death is via the deletion of the differentiation-inducing *PCDH11X* and *TGIF2LX*, which are also deleted in the brain tumors, and the amplification of the chromosome segregation checkpoint gene *MAD1L1*, whose amplification in the brain tumors is independently correlated with shorter survival. High levels of *MAD1L1* may protect such aneuploid stem-like cells from apoptosis on the cellular level.^77^ Recent evidence suggests, however, that on the organismal level, and early in embryonic development, the embryonic self-correction program may be able to deplete these cells from the embryonic germ layers spontaneously^78^ (i.e., without intervention).

By determining differential RNA expression that consistently maps to the DNA CNAs in the tumors (Figs. 12 and 13), the patterns predict response to existing FDA-approved therapies, as well as to new treatments that are being tested in clinical trials. For example, they describe orthogonal mechanisms of resistance to retinoic acid-induced differentiation. In $u_{1,1}$, the resistance is acquired through underexpression of the retinoic acid receptor *RARA*, together with its co-deletion with the embryonic morphogenesis regulator *SHH* and the neurogenic differentiation activator *NEUROD2*.^79^ In $u_{1,101}$, where *RARA* is overexpressed, it is through its co-amplification with the enzymes *CYP2W1* and *UGT2B17*, which convert retinoic acid into inactive compounds.^80,81^

Finally, by separating the two predictors from normal variations, the patterns identify disease-specific alterations that map to normally varying genomic features, including, for example, the differential co-alteration of *PCDH11X* and *TGIF2LX* on the X chromosome. The closest homologs to the only two genes that remain at the human male-specific X chromosome-transposed region on the Y chromosome after its three million years of evolution,^82^ *PCDH11X* and *TGIF2LX*, are also implicated in cellular responses to retinoic acid.^83^

Taken together, the two predictors describe independent, yet complementary, known and new disease mechanisms and druggable targets that transform normal human cells into tumor cells.

## DISCUSSION

We introduced a quantum mechanics-based multitensor AI/ML and showed that it uniquely can solve the challenges of real-world multiomic data that are small-cohort, noisy, and high-dimensional. We proved that the algorithms always converge to a model and that the model is almost always unique, whose computation is lossless by definition. We defined metrics necessary to derive, test, and explain the model.

We highlighted the novel connection of this AI/ML to the two fundamental principles at the mathematical foundation of quantum mechanics—entanglement and superposition: The model rewrites a set of multiple omic profiles from one patient as a superposition of phenotypes, each represented by a set of multiple entangled patterns. This means that the prediction of a phenotype based upon the measurement of any one omic profile approximately determines the result of the measurement of any other profile. The predictors encompass the whole multiome, and that is one reason why, in all available examples, they outperform all other biomarkers where they exist.

We illustrated the AI/ML in the discovery, validation, and interpretation of two novel predictors in NBL—each with three entangled representations—in the tumor and blood genomes and the tumor transcriptome, where we showed that entanglement holds: The determination of patients’ survival and treatment responses via the measurement of any one representation is consistent with the measurements of the remaining two. In every representation, the two predictors are consistently more accurate than the best standard-of-care biomarker (i.e., the one-gene test for tumor *MYCN* amplification). In addition, the interpretation of any one representation, in terms of known and new disease mechanisms and drug targets, coherently complements the interpretations of the remaining two. One predictor includes co-amplification of the known *MYCN* with druggable targets previously unrecognized in NBL (e.g., X chromosome-mapped genes and genes that encode for extra-embryonic transcripts). The other includes the known *ID2* amplification and describes an earlier stage in NBL development, where the embryonic program is hijacked toward aneuploidy and where subsequent tumor development can spontaneously regress via embryonic self-correction.

Our predictors are more accurate, actionable, and interpretable than other recent NBL models. At 73%–80% concordance with survival, our two NBL predictors combined outperform a black-box neural network, which itself surpassed the one-matrix principal component analysis (PCA) and other clustering methods by reaching 71% concordance with survival after selecting 100 features from NBL genomes and transcriptomes.^84^ By identifying new disease mechanisms and drug targets in addition to most of the known ones, including both *MYCN* and *ID2*, the two predictors also outperform three recent non-prognostic signatures: One catalog of somatic mutations in cancer (COSMIC) signature was derived from 23 829 cancer patient samples by using a one-matrix non-negative matrix factorization (NMF) and interpreted by using the genomes of 1019 additional NBL patient samples.^85^ The other two were derived from NBL single-cell transcriptomes.^86,87^ Each of these signatures identified either *MYCN* or *ID2*, but none identified both.

The new drugs that our predictors identified are consistent with existing evidence, yet functional genomic analyses of these genes would be critical, and we have previously successfully performed such experiments in other cancers. For example, our recent CRISPR-Cas9 experiments validated our predictions of a target to sensitize GBM tumors as well as the tumors’ differential responses to its targeting^10,56^ (also Ref. 57).

We conclude that our quantum mechanics-based multitensor AI/ML is uniquely able to discover, validate, and interpret predictors from small-cohort, noisy, and high-dimensional multiomic data.

## Supplementary Material

Johnson-Groh_AIP_Publishing_2026_Feature.pdf

## Figures and Tables

**TABLE I.** The GSVD predictors combined are statistically better than and independent of all standard-of-care indicators. Among the 90 patients, the standard indicators correspond to shorter median survival differences and greater log-rank *P*-values than the predictors, derived from $u_{1,1}$ and $u_{1,101}$ across the 2 831 960 tumor DNA features, together.

Patients (number)	Predictor/indicator	KM group	Median (months)	Log-rank *P*-value	Cox model	Hazard ratio	Hazard interval	Wald *P*-value	Concordance index	AIC

90	Tumor DNA 1	Low	—	6.0 × 10^−4^	Univariate	2.9	1.5–5.4	1.0 × 10^−3^	0.77	334
		High	31							
	
	Tumor DNA 101	Low	—	2.3 × 10^−4^		3.1	1.6–5.7	4.5 × 10^−4^	0.74	331
		High	48							

90	Tumor DNA 1	Low	—	8.5 × 10^−5^	Bivariate	2.3	1.2–4.4	1.1 × 10^−2^	0.75	327
	
	Tumor DNA 101	Low/high	60			2.6	1.4–5.0	3.2 × 10^−3^		
		High/low	40							
		High	26							

90	Tumor DNA 1+101	Low	—	2.3 × 10^−5^	Univariate	4.0	2.0–8.1	8.6 × 10^−5^	0.80	326
		High	48							
	
	INSS stage	4S	—	2.1 × 10^−3^		3.3	1.5–7.3	3.3 × 10^−3^	0.83	327
		<4								
		4	67							
	
	Age (months)	<18	—	2.2 × 10^−3^		4.4	1.6–12.3	5.0 × 10^−3^	0.76	332
		≥18	68							
	
	*MYCN* amplification	No	—	5.7 × 10^−3^		2.4	1.3–4.5	7.3 × 10^−3^	0.73	337
		Yes	37							
	
	Histopathology	F	—	1.3 × 10^−3^		5.6	1.7–18.0	4.3 × 10^−3^	0.81	330
		U	68							
	
	DNA ploidy	H	—	3.7 × 10^−4^		3.0	1.6–5.5	6.8 × 10^−4^	0.75	332
		D	55							
	
	COG risk	L	—	7.7 × 10^−3^		3.2	1.3–7.5	8.2 × 10^−3^	0.80	331
		I								
		H	68							
	
	MKI	L	—	1.3 × 10^−4^		2.4	1.6–3.7	5.8 × 10^−5^	0.76	326
		I	91							
		H	37							

90	Tumor DNA 1+101	Low/<4	—	9.6 × 10^−5^	Bivariate	3.0	1.5–6.0	2.7 × 10^−3^	0.81	319
	
	INSS stage	Low/4S				2.7	1.2–5.9	1.7 × 10^−2^		
		Low/4								
		High/4S								
		High/4	44							
		High/<4	40							
	
	Tumor DNA 1+101	Low/<18	—	8.6 × 10^−5^		3.2	1.5–6.5	1.6 × 10^−3^	0.78	323
	
	Age (months)	Low/≥18				2.9	1.0–8.5	4.7 × 10^−2^		
		High/<18								
		High/≥18	48							
	
	Tumor DNA 1+101	Low/no	—	5.6 × 10^−5^		3.6	1.7–7.3	5.6 × 10^−4^	0.77	326
	
	*MYCN* amplification	Low/yes				. . .	. . .	. . .		
		High/no	60							
		High/yes	30							
	
	Tumor DNA 1+101	Low/F	—	2.6 × 10^−5^		3.1	1.5–6.3	1.8 × 10^−3^	0.79	321
	
	Histopathology	Low/U				3.8	1.1–12.5	3.1 × 10^−2^		
		High/F								
		High/U	44							
	
	Tumor DNA 1+101	Low/H	—	3.5 × 10^−5^		3.1	1.5–6.6	2.8 × 10^−3^	0.77	324
	
	DNA ploidy	Low/D				. . .	. . .	. . .		
		High/H	78							
		High/D	37							
	
	Tumor DNA 1+101	Low/L	—	5.5 × 10^−4^		3.1	1.5–6.4	1.7 × 10^−3^	0.79	322
	
	COG risk	Low/I				2.3	1.0–5.4	4.7 × 10^−2^			
		Low/H									
		High/L									
		High/H	48								
		High/I	16								
	
	Tumor DNA 1+101	Low/L	—	1.6 × 10^−5^		2.9	1.4–6.1	4.8 × 10^−3^	0.77	319
	
	MKI	Low/I				1.9	1.2–2.9	5.4 × 10^−3^		
		High/L								
		Low/H								
		High/I	44							
		High/H	31							

**TABLE II.** The multiomic representations of the predictors are entangled: Consistent determination of the patients’ phenotypes from their tumor or blood genotypes or tumor transcriptypes. In the classifications of the 90 patients by the representations of the GSVD predictors $u_{2,1}$ and $u_{2,101}$ across the 2 831 959 WGS blood DNA features; the 62 of the 71 patients by the corresponding HO GSVD predictors $u_{3,4}$ and $u_{3,2}$ across the 15 393 tumor RNA features; and the 398 of the 419 validation patients by the GSVD predictors $u_{1,1}$ and $u_{1,101}$ across the 10 354 tumor DNA features that are shared between the TCS and WGS profiles, the corresponding univariate Cox hazard ratios are consistently within the 95% confidence intervals of those of the GSVD predictors across the 2 831 960 WGS tumor DNA features and among the 90 discovery patients.

Patients (number)	Predictor	KM group	Median (months)	Log-rank *P*-value	Cox model	Hazard ratio	Confidence interval	Wald *P*-value	Concordance index	AIC

90	Blood DNA 1	Low High	— 32	3.0 × 10^−3^	Univariate	2.5	1.3–4.7	4.1 × 10^−3^	0.74	336
	
	Blood DNA 101	Low High	— 73	7.6 × 10^−3^		2.9	1.3–6.5	1.1 × 10^−2^	0.73	336

90	Blood DNA 1	Low	—	1.3 × 10^−3^	Bivariate	2.3	1.2–4.3	1.0 × 10^−2^	0.73	331
	
	Blood DNA 101	High/low				2.7	1.2–6.0	2.0 × 10^−2^		
		Low/high								
		High	31							

90	Blood DNA 1 + 101	Low High	— 73	6.8 × 10^−3^	Univariate	3.4	1.3–8.6	1.1 × 10^−2^	0.75	335

62	Tumor RNA 4	Low High	— 31	1.5 × 10^−2^	Univariate	2.9	1.2–7.0	2.0 × 10^−2^	0.77	182
	
	Tumor RNA 2	Low High	— 56	5.3 × 10^−5^		5.0	2.1–11.7	2.5 × 10^−4^	0.81	172

62	Tumor RNA 4	Low	—	1.9 × 10^−7^	Bivariate	3.0	1.2–7.5	1.9 × 10^−2^	0.80	169
	
	Tumor RNA 2	High/low				5.1	2.1–12.2	2.4 × 10^−4^		
		Low/high	78							
		High	19							

62	Tumor RNA 4+2	Low High	— 67	8.5 × 10^−4^	Univariate	4.0	1.7–9.8	2.0 × 10^−3^	0.79	176

398	Tumor DNA 1	Low High	—	2.8 × 10^−4^	Univariate	2.0	1.4–3.0	3.7 × 10^−4^	0.70	1172
	
	Tumor DNA 101	Low High	—	1.9 × 10^−6^		2.5	1.7–3.7	4.2 × 10^−6^	0.73	1164

398	Tumor DNA 1	Low	—	2.3 × 10^−5^	Bivariate	. . .	. . .	. . .	0.70	1165
	
	Tumor DNA 101	High/low				2.2	1.3– 3.9	3.4 × 10^−3^		
		High								
		Low/high	92							

398	Tumor DNA 1 + 101	Low High	—	4.8 × 10^−6^	Univariate	2.5	1.7–3.7	9.7 × 10^−6^	0.73	1164

**TABLE III.** In each of their entangled representations, the GSVD predictors combined are consistently more accurate than the best standard-of-care biomarker, that is, the one-gene test for tumor *MYCN* amplification. Among the 398 patients, *MYCN* amplification is at 70% concordance with survival, less than the 77% concordance of the combination of the GSVD predictors $u_{2,1}$ and $u_{2,101}$ across the 10 475 blood DNA features that are shared between the TCS and WGS profiles.

Patients (number)	Predictor/indicator	KM group	Median (months)	Log-rank *P*-value	Cox model	Hazard ratio	Confidence interval	Wald *P*-value	Concordance index	AIC

398	Blood DNA 1	Low	—	1.3 × 10^−3^	Univariate	2.7	1.4–5.0	2.0 × 10^−3^	0.74	1172
		High								
	
	Blood DNA 101	Low	—	2.1 × 10^−3^		1.9	1.3–2.9	2.5 × 10^−3^	0.68	1176
		High								

398	Blood DNA 1	Low	—	4.8 × 10^−4^	Bivariate	2.5	1.3–4.7	4.6 × 10^−3^	0.70	1168
	
	Blood DNA 101	Low/high				1.7	1.1–2.6	1.0 × 10^−2^		
		High/low								
		High								

398	Blood DNA 1+101	Low	—	1.3 × 10^−3^	Univariate	2.9	1.5–5.8	2.2 × 10^−3^	0.77	1172
		High								
	
	INSS stage	4S	—	1.6 × 10^−14^		5.8	3.3–9.9	3.5 × 10^−10^	0.86	1121
		<4								
		4								
	
	Age (months)	<18	—	2.5 × 10^−6^		2.7	1.8–4.2	6.2 × 10^−6^	0.70	1162
		≥18								
	
	*MYCN* amplification	No	—	1.6 × 10^−4^		2.1	1.4–3.1	2.2 × 10^−4^	0.70	1172
		Yes								
	
	Histopathology	F	—	2.6 × 10^−13^		7.3	3.9–13.7	4.5 × 10^−10^	0.87	1122
		U								
	
	DNA ploidy	H	—	7.7 × 10^−7^		2.6	1.7–3.8	1.9 × 10^−6^	0.73	1163
		D								
	
	COG risk	L	—	5.4 × 10^−15^		6.0	3.4–10.7	1.4 × 10^−9^	0.90	1103
		I								
		H								
	
	MKI	L	—	5.1 × 10^−7^		1.9	1.5–2.4	2.9 × 10^−7^	0.72	1157
		I								
		H								

398	Blood DNA 1+101	Low/<4	—	2.3 × 10^−14^	Bivariate	2.5	1.3–5.0	8.3 × 10^−3^	0.84	1114
	
	INSS stage	Low/4S				5.6	3.2–9.6	8.2 × 10^−10^		
		High/<4								
		High/4S								
		Low/4								
		High/4								
	
	Blood DNA 1+101	Low/<18	—	1.1 × 10^−6^		2.7	1.4–5.3	4.7 × 10^−3^	0.71	1153
	
	Age (months)	Low/≥18				2.6	1.7–4.0	1.6 × 10^−5^		
		High/<18								
		High/≥18								
		
397	Blood DNA 1+101	Low/no	—	6.7 × 10^−5^		2.3	1.1–4.7	2.1 × 10^−2^	0.71	1166
	
	*MYCN* amplification	High/no				1.8	1.2–2.7	5.6 × 10^−3^		
			High/yes							

398	Blood DNA 1+101	Low/F	—	2.1 × 10^−13^	Bivariate	2.4	1.2–4.7	1.3 × 10^−2^	0.84	1117
	
	Histopathology	High/F				6.9	3.7–12.9	1.6 × 10^−9^		
		Low/U								
		High/U								
	
	Blood DNA 1+101	Low/H	—	4.2 × 10^−7^		2.6	1.3–5.1	7.4 × 10^−3^	0.74	1156
	
	DNA ploidy	Low/D				2.4	1.6–3.5	1.3 × 10^−5^		
		High/H								
		High/D								
	
	Blood DNA 1+101	Low/L	—	5.1 × 10^−14^		2.0	1.0–4.0	4.4 × 10^−2^	0.87	1100
	
	COG risk	High/L				5.6	3.2–10.1	5.1 × 10^−9^		
		Low/I								
		High/I								
		Low/H								
		High/H								
	
	Blood DNA 1+101	Low/L	—	3.9 × 10^−7^		2.5	1.3–5.1	7.8 × 10^−3^	0.73	1150
	
	MKI	High/L				1.8	1.4–2.3	1.7 × 10^−6^		
		Low/I								
		Low/H								
		High/I								
		High/H								

## Data Availability

Datasets 1–3 are available upon request in TXT format. **Dataset 1:** Clinical, sample, and profile labels of the discovery set of X=101 NBL patients, reproduced from the TARGET project. Also available are the corresponding tumor and blood DNA profiles, tabulating the log_2_ of the Complete Genomics-measured DNA sequence read counts in $Z_{1}=2\;831\;960$ and $Z_{2}=2\;831\;959$ nonoverlapping 1K-nucleotide bins across the autosome and the X chromosome of the hg19 reference human genome, where each profile is centered at its autosomal median, and the tumor RNA profiles of 71 of the patients, tabulating the log_2_ of the Illumina Hi-Seq 2000-measured RNA read counts of $Z_{3}=15\;393$ transcripts. **Dataset 2:** Clinical, sample, and profile labels of the validation set of Y=419 NBL patients. Also available are the corresponding tumor and blood DNA profiles, tabulating the Illumina Hi-Seq 2500-measured DNA read counts in the $Z_{1}=10\;354$ and $Z_{2}=10\;475$ bins that are shared with the discovery WGS tumor and blood DNA bins, respectively. **Dataset 3:** Genomic, segmentation, and CNA labels of the CBS segments in the GSVD tumor DNA-specific patterns $u_{1,k}$, k=1,100,101. Mathematica Notebook 1 is available upon request in PDF format.
