# Supplementary material for: Quantum mechanics-based multitensor AI/ML uniquely able to discover, validate, and interpret predictors from small-cohort noisy high-dimensional multiomic data
Source: APL Quantum. Author manuscript; Available in PMC 2026 Sep 23. (PMC13596398; doi:10.1063/5.0305656)
Supplement: Supplementary file 1 [file NIHMS2199341-supplement-Johnson-Groh_AIP_Publishing_2026_Feature_pdf.pdf]

Scilight

NEWS | JUNE 22 2026

A quantum mechanics approach to artificial intelligence can improve cancer outcomes FREE

Mara Johnson-Groh

Check for updates

Scilight 2026, 261101 (2026)
https://doi.org/10.1063/10.0044179

View Online
Export Citation

Connected Content

This is a companion to: Quantum mechanics-based multitensor AI/ML uniquely able to discover, validate, and interpret predictors from small-cohort noisy high-dimensional multiomic data

Articles You May Be Interested In

Two-scale structure of the current layer controlled by meandering motion during steady-state collisionless driven reconnection

Phys. Plasmas (July 2004)

Single particle motion near an X point and separatrix

Phys. Plasmas (June 2004)

26 June 2026 16:39:56

AIP Publishing

Scilight

22 June 2026

A quantum mechanics approach to artificial intelligence can improve cancer outcomes

Mara Johnson-Groh

Using principles of superposition and entanglement, researchers develop a framework to tailor a patient's cancer treatment to their entire molecular background.

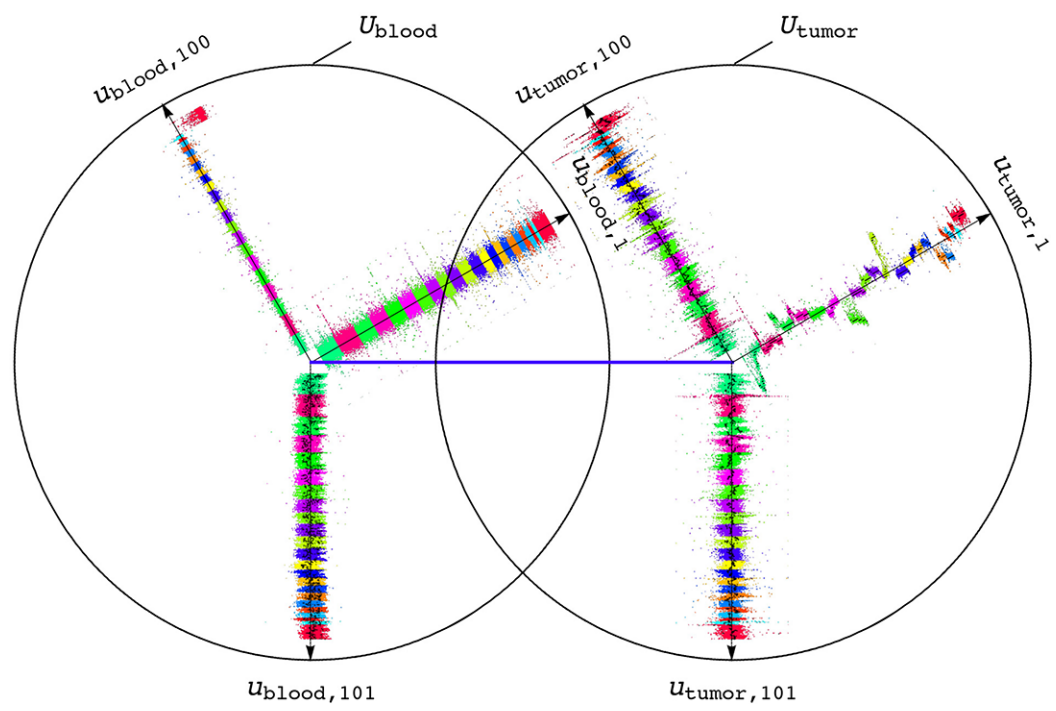

For a child diagnosed with neuroblastoma, the path to treatment isn't simple. Some types of neuroblastoma resolve on their own, while others require aggressive treatments. Researchers have tried matching treatments to patients based on one-gene mutations but with limited success, as patients' outcomes depend on their entire molecular background containing millions of features, such as DNA and RNA from tissues and blood. Current artificial intelligence and machine learning (AI/ML) approaches struggle to derive predictors from the 20-100 patient samples of a clinical trial because they require massive amounts of training data.

But by using the mathematics of quantum mechanics, Alter et al. developed an AI/ML technique that can improve treatment selections and drug success rates. The technique features a suite of algorithms, called multitensor comparative spectral decompositions, which were built on the quantum mechanical concepts of entanglement and superposition. They separate the molecular data from a patient into outcome components represented by patterns entangled across the feature types, similarly to how a prism splits white light into its color components.

The authors demonstrated their technique with an analysis of open-source data of neuroblastoma cases. Their algorithms discovered two new predictors, found in the tumor and blood genomes and tumor transcriptome, of patients' life expectancy in response to treatment that all other methods missed. The two predictors consistently outperformed standard biomarkers across tumor and blood DNA and tumor RNA.

"Neural network models are black boxes, but our predictors are interpretable; they point to disease mechanisms and suggest genes to target to sensitize tumors to treatment," said author Orly Alter. "Our team recently experimentally validated our predictions of adult glioblastoma patient outcome and drug targets in clinical trials and CRISPR-Cas9 studies."

Source: "Quantum mechanics-based multitensor AI/ML uniquely able to discover, validate, and interpret predictors from small-cohort noisy high-dimensional multiomic data," by Orly Alter, Elizabeth Newman, Sri Priya Ponnapalli, and Jessica W. Tsai, *APL Quantum* (2026). The article can be accessed at https://doi.org/10.1063/5.0305656.

Published by AIP Publishing (https://publishing.aip.org/authors/rights-and-permissions).

© Published by AIP Publishing

26 June 2026 16:39:56
